# Elemental composition control of gold-titania nanocomposites by site-specific mineralization using artificial peptides and DNA

**DOI:** 10.1038/s42004-020-00440-8

**Published:** 2021-01-04

**Authors:** Makoto Ozaki, Takahito Imai, Takaaki Tsuruoka, Shungo Sakashita, Kin-ya Tomizaki, Kenji Usui

**Affiliations:** 1 Faculty of Frontiers of Innovative Research in Science and Technology (FIRST), Konan University, 7-1-20 Minatojima-minamimachi, Chuo-ku, Kobe, 650-0047 Japan; 2 Department of Materials Chemistry, Ryukoku University, 1-5 Yokotani, Seta Oe-cho, 520-2194 Otsu, Japan; 3 Department of Materials Chemistry and Innovative Materials and Processing Research Center, Ryukoku University, 1-5 Yokotani, Seta Oe-cho, 520-2194 Otsu, Japan

**Keywords:** Organic-inorganic nanostructures, Nanoparticle synthesis, Peptides, Nanoparticles, Nanoparticles

## Abstract

Biomineralization, the precipitation of various inorganic compounds in biological systems, can be regulated in terms of the size, morphology, and crystal structure of these compounds by biomolecules such as proteins and peptides. However, it is difficult to construct complex inorganic nanostructures because they precipitate randomly in solution. Here, we report that the elemental composition of inorganic nanocomposites can be controlled by site-specific mineralization by changing the number of two inorganic-precipitating peptides bound to DNA. With a focus on gold and titania, we constructed a gold-titania photocatalyst that responds to visible light excitation. Both microscale and macroscale observations revealed that the elemental composition of this gold-titania nanocomposite can be controlled in several ten nm by changing the DNA length and the number of peptide binding sites on the DNA. Furthermore, photocatalytic activity and cell death induction effect under visible light (>450 nm) irradiation of the manufactured gold-titania nanocomposite was higher than that of commercial gold-titania and titania. Thus, we have succeeded in forming titania precipitates on a DNA terminus and gold precipitates site-specifically on double-stranded DNA as intended. Such nanometer-scale control of biomineralization represent a powerful and efficient tool for use in nanotechnology, electronics, ecology, medical science, and biotechnology.

## Abstract (web summary)

Biomineralization allows for crystal structure, size, and morphology control over inorganic compounds, but precipitation at random compositions hinders the construction of complex nanostructures. Here, the elemental composition of a gold-titania nanocomposite photocatalyst is controlled through two inorganic precipitating peptides bound to DNA.

## Introduction

Inorganic nanocomposites have been used in diverse applications in nanotechnology^1–3^, electronics^4–6^, and biotechnology^7,8^. However, conventional production methods such as hydrothermal synthesis and chemical reduction have some problems, as follows: 1) these conventional methods do not offer easily controlled size and structure; 2) it is difficult for these methods precisely control the elemental composition of inorganic nanocomposites; 3) these methods generally consume a considerable amount of energy (high temperature and high pressure); and 4) environmentally hazardous substances such as reducing agents, strong acids, and strong bases are often used. Mimicking biological systems is one of the most powerful approaches to overcome these problems. For example, in biomineralization systems, some proteins can be used to precipitate inorganic compounds. The function of such a system is to produce inorganic compounds on organic compounds such as proteins and peptides^9–19^. Inorganic compounds formed by mineralization are referred to as biominerals. Biominerals include teeth, bone, crustacean exoskeletons, pearls, shells, and the magnetosomes of magnetotactic bacteria and possess flexibility because of their hybrid structures, high mechanical strength, and optical properties because of their density. The reaction conditions for the production of biominerals are much milder than those used in conventional material synthesis. Therefore, such inorganic compounds formed by controlling mineralization at the nanometer level have a low environmental load and reduced energy consumption.

However, although in conventional mineralization solves problems 3) and 4) mentioned above 1) and 2) remain (i.e., because inorganic compounds precipitate randomly in solution, conventional mineralization cannot offer precise control of the structure, distribution, and elemental composition of inorganic nanocomposites). To solve these remaining problems, we aimed to prepare inorganic-precipitating molecules corresponding to each inorganic constituent of the nanocomposites and then precisely distribute these molecules onto well-shaped template molecules. Furthermore, the degree of distribution of each inorganic-precipitating molecule should be controlled. Finally, mineralization using these molecules is expected to produce inorganic nanocomposites with various elemental compositions. With these aims in mind, we applied to nanometer-scale site-specific mineralization to attempt to solve problems 1) and 2) using peptides as inorganic-precipitating molecules and DNA as well-shaped template molecules.

Peptides are promising compounds for use in nanocontrolled mineralization because they confer several advantages: (1) Small peptides derived from the sequence of isolated proteins for mineralization or artificial sequences can precipitate many types of inorganic compounds^9–19^. (2) Peptides can be conjugated with functional groups and molecules other than amino acids^20–24^, and such functionalized peptides can be distributed on well-shaped organic nanostructures that serve as templates for mineralization, such as DNA, which is used as DNA origami. (3) Peptides are easier to design and handle than proteins^25–29^.

We have previously constructed a simple nanometer-scale site-specific precipitation system using template DNA and an artificial peptide^30^. We have succeeded in precisely controlling the distribution of inorganic compounds. However, nanometer-scale site-specific mineralization by controlling two inorganic compounds using a peptide and DNA was difficult^31^. The site-specific mineralization of two inorganic compounds is expected to be controlled using two inorganic-precipitating peptides corresponding to each inorganic compound on the template DNA. Therefore, the elemental composition of the inorganic nanocomposites is expected to be controlled at the nanometer level by changing the number of peptides bound to the template DNA using our nanometer-scale site-specific mineralization method.

Meanwhile, titania materials with excellent photocatalytic activity are promising as one of the best environmental clean-up reagents. However, titania exhibits photocatalytic activity upon excitation only in the UV range^32–34^. Nanometer-sized gold is known to function as a co-catalyst with the titania photocatalyst^35–39^ and shows absorption derived from surface plasmon resonance (SPR) at approximately 520 nm. Therefore, a gold-titania photocatalyst is expected to exhibit photocatalytic activity under visible light irradiation and photoinduced charge separation.

**Fig. 1:**
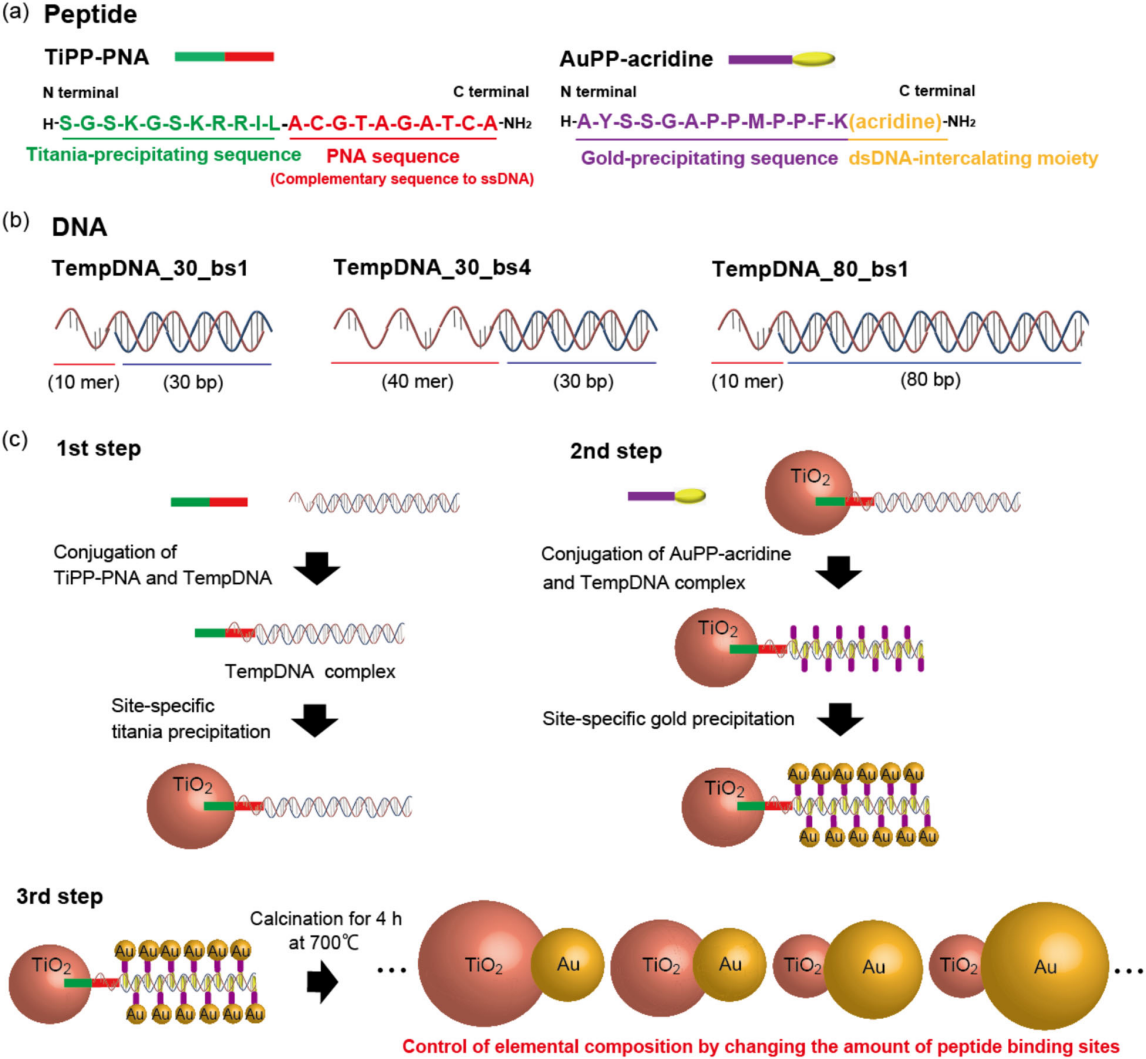
Design of peptides and DNAs. **a** Sequences of the titania-precipitating peptide (TiPP-PNA) and gold-precipitating peptide (AuPP-acridine) used in this study. **b** Illustration of the three DNA sequences (TempDNA_30_bs1, TempDNA_30_bs4, and TempDNA_80_bs1) used in this study (the other three DNA sequences are shown in Supplementary Fig. 2). **c** Outline of the site-specific titania and gold precipitation using the DNA and the two peptides (TiPP-PNA and AuPP-acridine).

Here we report that the elemental composition of gold-titania nanocomposites can be controlled by changing the number of two inorganic precipitating peptides bound to DNA (Fig. 1c). Furthermore, we attempted to construct gold-titania nanocomposites that respond to visible light excitation by nanometer-scale site-specific mineralization changing the contents of titania and gold. In nanotechnology and electronics fields, our proposed method for site-specific precipitation can be expected to produce multimetallic catalysts with high catalytic activity and alloy materials with high electrical conductivity, and so on. In biotechnology and medical field, our proposed method can be expected to produce Q-dot with fluorescence quantum yield for in vivo imaging and carriers in photothermal therapy of cancer cells, and so on. Therefore, our method thus represents a powerful and efficient technique for use in nanotechnology, electronics, and biotechnology.

## Results and Discussion

### Design and synthesis of peptides and DNA

We first designed the peptides using Fmoc solid-phase synthesis (See Supplementary Methods 1 in Supplementary information)^40,41^. The titania-precipitating peptide (TiPP-PNA) and gold-precipitating peptide (AuPP-acridine) used in this study both consisted of two parts (Fig. 1a). TiPP-PNA consisted of a previously described titania-precipitating peptide sequence (TiPP, Supplementary Fig. 1a)^42^ and a PNA sequence that was used to bind to a complementary DNA sequence (Fig. 1b and Supplementary Fig. 2)^30,31^. AuPP-acridine consisted of a previously described gold-precipitating peptide sequence (AuPP, Supplementary Fig. 1b)^43^ and acridine, which was used to intercalate into double-stranded DNA^24^. We confirmed that these peptides were synthesized in high purity (Supplementary Fig. 3a–d). The DNA used in this study consisted of two parts: single-stranded for the TiPP-PNA binding site and a DNA duplex for AuPP-acridine intercalation. One molecule of acridine intercalates per three DNA bases^24^. Consequently, we expected to control the elemental composition of the gold-titania nanocomposite by changing the duplex DNA length to modify AuPP-acridine binding and the single-stranded DNA length (and thus the number of PNA binding sites) to modify TiPP-PNA binding.

The following DNA sequences were designed (See Fig. 1b, Table 1, Supplementary Fig. 2, and Supplementary Methods 2 in Supplementary information): TempDNA_30_bs1, which was a 30 bp DNA (approximately 10 nm) with 1 PNA binding site at the 5′ terminus and approximately 10 acridine intercalating sites at the double strand; TempDNA_30_bs4, which was a 30 bp DNA (approximately 10 nm) with 4 PNA binding sites at the 5′ terminus and approximately 10 acridine intercalating sites at the double strand; TempDNA_80_bs1, which was an 80 bp DNA (approximately 30 nm) with 1 PNA binding site at the 5′ terminus and approximately 20 acridine intercalating sites at the double strand; TempDNA_150_bs1, which was a 150 bp DNA (approximately 50 nm) with 1 PNA binding site at the 5′ terminus and approximately 50 acridine intercalating sites at the double strand; TempDNA_300_bs1, which was a 300 bp DNA (approximately 100 nm) with 1 PNA binding site at the 5′ terminus and approximately 100 acridine intercalating sites at the double strand; TempDNA_600_bs1, which was a 600 bp DNA (approximately 200 nm) with 1 PNA binding site at the 5′ terminus and approximately 200 acridine intercalating sites at the double strand. By using these DNAs and the two peptides in mineralization (Fig. 1c) gold nanoparticle and titania nanoparticle would be bound to the DNA and these peptides could fully occupy the binding sites of DNA (Supplementary Table 1). Then gold-titania nanocomposite with various gold and titania composition would be generated by calcination and would show various high photocatalytic activity under visible light irradiation depend on gold and titania composition due to the transfer of electrons from gold to titania.

**Table 1:** Properties of manufactured gold-titania nanocomposites by site-specific mineralization using various TempDNAs.

Properties	TempDNA_30_bs4	TempDNA_30_bs1	TempDNA_80_bs1	TempDNA_150_bs1	TempDNA_300_bs1	TempDNA_600_bs1
Structure size^a^	133.5 nm	111.8 nm	147.0 nm	n.d.^b^	n.d.^b^	n.d.^b^
Au/Ti^c^ (Content ratio)	2.1	2.7	4.0	n.d.^b^	n.d.^b^	n.d.^b^
TiPP-PNA binding sites	4	1	1	1	1	1
AuPP-acridine intercalating sites	10	10	20	50	100	200

^a^Structure sizes are measured by DLS.

^b^Gold with bulk size was formed, and it was impossible to fabricate the gold-titania nanocomposite.

^c^The content ratio were measured by ICP-AES.

### Control of the elemental composition of gold-titania nanocomposite

After optimizing the reaction conditions of titania precipitation (See Supplementary Methods 3 in Supplementary information) and gold precipitation (See Supplementary Methods 4 in Supplementary information), we constructed gold-titania nanocomposites by a nanometer-scale site-specific precipitation method, as shown in Fig. 1c. Furthermore, we confirmed using macro-scale observations, such as inductively coupled-plasma atomic emission spectroscopy (ICP-AES, See Supplementary Methods 5 in Supplementary information) and dynamic light scattering (DLS, See Supplementary Methods 6 in Supplementary information), that the elemental composition and size of the gold-titania nanocomposites could be controlled by changing the length of the DNA and the number of PNA binding sites on the DNA.

The gold and titanium composition of the gold-titania nanocomposite was identified using ICP-AES. The content ratio of gold and titanium (amount of gold to titanium) in the gold-titania nanocomposites produced with TempDNA_30_bs4 was 2.1, that for TempDNA_30_bs1 was 2.7, and that for TempDNA_80_bs1 was 4.0 (Table 1 and Supplementary Table 1). These results showed that the elemental composition of the gold-titania nanocomposites can be controlled by changing the DNA length and the number of PNA binding sites on the DNA molecule. We also used longer DNA (TempDNA_150_bs1, TempDNA_300_bs1, and TempDNA_600_bs1), but gold with a bulk size was formed and it was impossible to fabricate the gold-titania nanocomposite (Table 1 and Supplementary Table 1). Next, the size of the gold-titania nanocomposite that could be produced using different DNA templates (TempDNA_30_bs1, TempDNA_30_bs4, and TempDMA_80_bs1) was identified using DLS. TempDNA_30_bs1, TempDNA_30_bs4, and TempDNA_80_bs1 showed nanocomposites of expected sizes upon DLS analysis (i.e., TempDNA_30_bs1 showed nanocomposites of approximately 111.8 nm, TempDNA_30_bs4 showed nanocomposites of approximately 133.5 nm, and TempDNA_80_bs1 showed nanocomposites of approximately 147.0 nm; Table 1). These macroscopic analyses showed that the elemental composition of the gold-titania nanocomposites can be controlled by changing DNA lengths and the number of PNA binding sites.

**Fig. 2:**
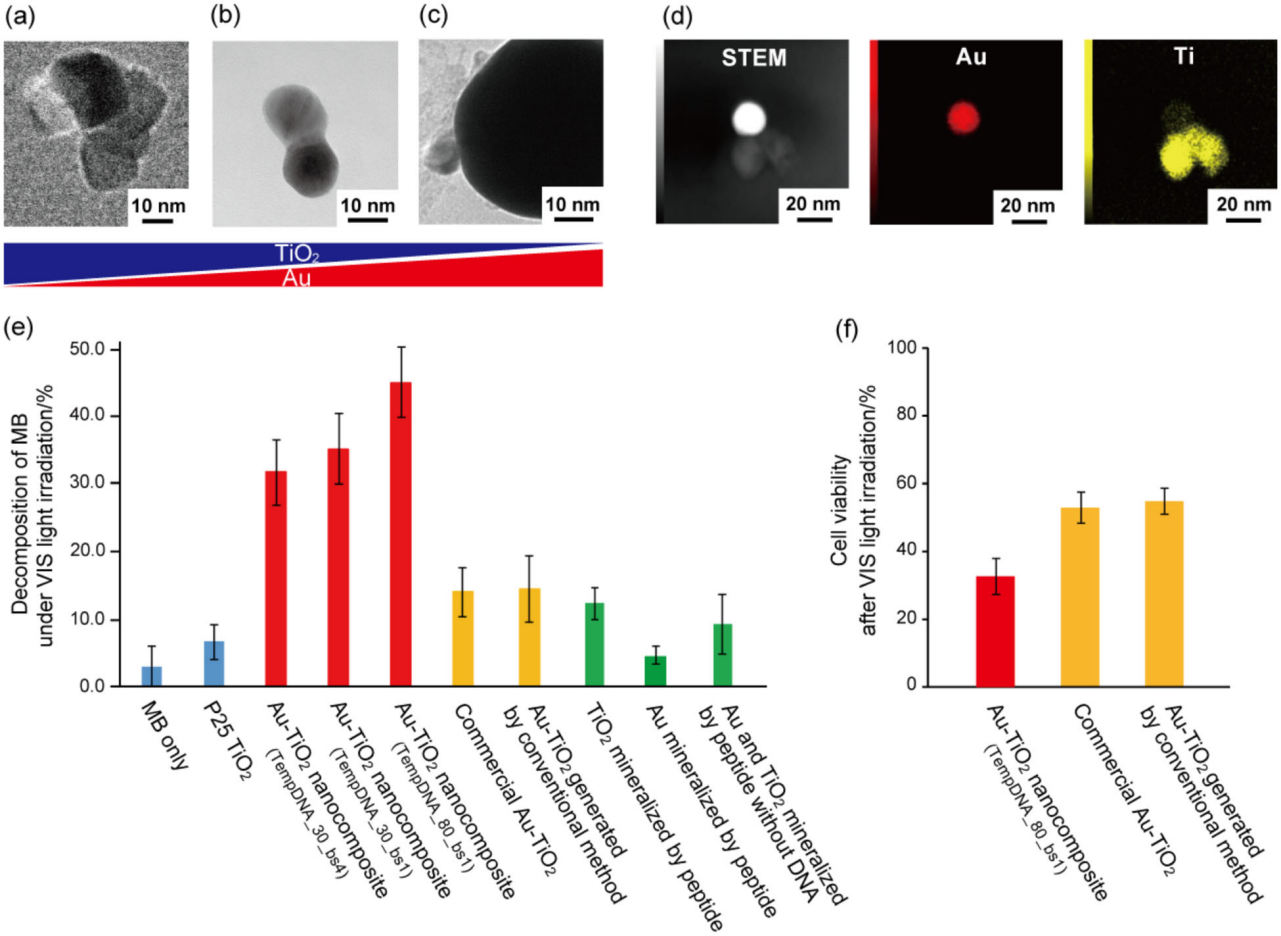
Elemental composition control and of gold-titania nanocomposites and photocatalytic activity evaluation under visible light irradiation. TEM images of gold-titania nanocomposites with **a** TempDNA_30_bs4, **b** TempDNA_30_bs1, and **c** TempDNA_80_bs1. **d** TEM-EDX mapping images of gold-titania nanocomposite with TempDNA_30_bs4. **e** Decomposition of MB under visible light (>450 nm) irradiation using various titania, gold, and gold-titania samples. Results are the mean ± SEM, *n* = 3. **f** Cell death induction effect under visible light (>450 nm) irradiation of various gold-titania samples. Results are the mean ± SEM, *n* = 3.

We also conducted transmission electron microscopy (TEM) analyses (See Supplementary Methods 7 in Supplementary information) to determine whether the results would be correlated with those obtained with ICP-AES and DLS data. The nanocomposites consisting of a titania nanoparticle of approximately 20 nm and a gold nanoparticle of approximately 20 nm were observed with TempDNA_30_bs1 (Fig. 2b and Supplementary Fig. 4b). The nanocomposites consisting of a titania nanoparticle of approximately 20 nm similar to that of TempDNA_30_bs1 and a gold nanoparticle of approximately 90 nm were observed with TempDNA_80_bs1 (Fig. 2c and Supplementary Fig. 4c). Furthermore, the nanocomposites consisting of a titania nanoparticle of approximately 50 nm (aggregate of 1 to 4 titania particles of approximately 20 nm) and a gold nanoparticle of approximately 20 nm similar to that of TempDNA_30_bs1 were observed with TempDNA_30_bs4 (Fig. 2a and Supplementary Fig. 4a). We then determined the localization of gold and titania by TEM-EDX and observed titanium and gold in the nanocomposite. These images suggested that gold and titania were precisely distributed site-specifically on DNA as we designed (Fig. 1 and Fig. 2d). These results showed that the distribution of titania and gold could be controlled in several ten nm range by mineralization. The manufactured gold-titania nanocomposites did not collapse even after calcination. We considered that gold and titania formed composite structure by mineralization to some extent before the calcination. Then, the structure of gold-titania nanocomposites was maintained after calcination. In addition, we considered that the burnout of peptide-DNA complex delayed because the complex was located inside the gold-titania nanocomposite. Furthermore, these results also suggested that the gold and titania particle sizes of gold-titania nanocomposites correlated with amount of gold and titanium by ICP-AES. These macro-scale observation and micro-scale observation results showed that the elemental composition of the gold-titania nanocomposite could be controlled successfully by changing the DNA length and the number of PNA binding sites on DNA and the peptide concentrations despite the same preparation conditions, such as the concentrations of titanium bis(ammonium lactato)dihydroxyde (TiBALDH) and HAuCl_4_.

### Photocatalytic activity under visible light irradiation of gold-titania nanocomposites

The photocatalytic activity under visible light irradiation was controlled by changing the content ratio of gold-titania nanocomposites. The photocatalytic activity of the gold-titania nanocomposite was evaluated by the degradation of methylene blue (MB)^44^ under visible light irradiation (>450 nm, to avoid titania absorption), which was measured by the change in the main absorption peak of MB at 665 nm (Fig. 2e). The photocatalytic activity evaluation of these nanocomposites was performed under the same amount of titanium contents in each sample (0.1 mg titania in a sample of photocatalytic activity measurements) based on ICP-AES data (Table 1). MB discoloration was not observed in the control sample (MB only). Commercial titania, P25 TiO_2_ (Nippon Aerosil Co. Ltd., Tokyo, Japan; tiania nanoparticle of 21 nm with mixed phases of anatase and rutile; Supplementary Fig. 5), showed little photocatalytic activity under visible light irradiation. The gold-titania nanocomposite prepared with TempDNA showed high photocatalytic activity under visible light irradiation. In addition, as the elemental composition of gold in the gold-titania nanocomposite increased, the photocatalytic activity increased. The photocatalytic activity under visible light irradiation of the gold-titania nanocomposite produced with TempDNA_80_bs1 was higher than that of the gold-titania nanocomposites produced with TempDNA_30_bs4 and TempDNA_30_bs1, and additionally that with TempDNA_30_bs1 was slightly higher than that with TempDNA_30_bs4 (Fig. 2e). These results suggest that the charge transfer efficiency was improved by an increase in the gold content in the gold-titania nanocomposite, which increased the photocatalytic activity. These results showed that the photocatalytic activity could be controlled by changing the gold content in the gold-titania nanocomposite. Furthermore, we evaluated the photocatalytic activity under visible light irradiation (>450 nm) of commercial gold-titania (RR2Ti, Haruta Gold Co. Ltd., Tokyo, Japan; The amount of gold supported on titania is 0.96 wt%; Supplementary Fig. 6a, b) and gold-titania generated by conventional method^45^ (the preparation ratio of Au and Ti is 4:1; See Supplementary Methods 8 and Supplementary Fig. 7) as the negative control samples. Both the photocatalytic activities of commercial gold-titania and gold-titania generated by conventional method were more than three times lower than that of mineralized gold-titania nanocomposite with TempDNA_80_bs1 (Fig. 2e). These results indicated that gold-titania nanocomposite generated by our site-specific mineralization method had high photocatalytic activity under visible light irradiation. On the basis of the above results, we decided to perform various analyses using TempDNA_80_bs1 because the gold-titania nanocomposite with TempDNA_80_bs1 showed the highest photocatalytic activity. The photocatalytic activity under visible light irradiation can be successfully controlled by changing the gold and titania contents in the gold-titania nanocomposite using our site-specific mineralization method.

### Cytotoxicity of gold-titania nanocomposites

Additionally, we confirmed by cytotoxicity tests (See Supplementary Methods 9 and 10 in Supplementary information) that the gold-titania nanocomposites manufactured using our nanometer-scale site-specific mineralization method is not toxic, although because titania produced by conventional methods using environmentally hazardous substances such as reducing agents, strong acids, and strong bases is toxic. Titania is coated on the surface of building materials and glass to facilitate the decomposition of organic compounds such as dirt. Therefore, photocatalysts are required to have no cytotoxicity. Thus, we conducted a cytotoxicity test of gold-titania nanocomposite with TempDNA_80_bs1, commercial gold-titania, and gold-titania generated by the conventional method, which were also used in the photocatalytic activity evaluation (Fig. 2e). The cell viabilities evaluated by Cell Counting Kit-8 (CCK-8) assay were 99.1% for gold-titania nanocomposite with TempDNA_80_bs1, 97.3% for commercial gold-titania, and 98.2% for gold-titania generated by a conventional method. These results suggested that the gold-titania nanocomposite was not cytotoxic. Biocompatible gold-titania nanocomposites can be constructed by our nanometer-scale site-specific mineralization, which does not use environmentally hazardous substances.

### Cell death induction effect under visible light irradiation of gold-titania nanocomposites

TiO_2_ produces radicals under UV light irradiation, which exerts oxidative stress on cells and induces cell death. Thus, we evaluated cell death induction effects (cytotoxic effects) of gold-titania samples under visible light irradiation using HeLa cells (See Supplementary Methods 9 and 11 in Supplementary information). Gold-titania samples of 0.1 mg/mL were added to HeLa cells, and HeLa cells were irradiated with visible light (>450 nm) for 20 min. The cell viabilities after irradiation with visible light for 20 min were 32.7 ± 5.3% for gold-titania nanocomposite with TempDNA_80_bs1, 52.9 ± 4.6% for commercial gold-titania, and 54.9 ± 3.9% for gold-titania generated by conventional method (Fig. 2f). These results suggested that gold-titania nanocomposite using our method, which showed higher photocatalytic activity under visible light irradiation, showed higher cell death induction effect than those with conventional methods.

### Site-specific precipitation of gold on double-stranded DNA

The gold precipitation conditions using AuPP-acridine alone were observed using UV-VIS measurements by changing the precipitation time and sodium citrate concentration (See Supplementary Methods 12 in Supplementary information). For all sodium citrate concentrations, an increase in absorption derived from SPR at approximately 520–540 nm was evident over a precipitation time of 3 h by UV–VIS measurements (Supplementary Fig. 8). Next, the sizes of the gold nanoparticles were compared after gold precipitation with/without AuPP-acridine. UV–VIS measurements of the samples with AuPP-acridine showed no shift in the SPR absorbance peak (Supplementary Fig. 8); however, the absorbance peak derived from SPR shifted for the samples without AuPP-acridine (Supplementary Fig. 8). The absorbance peak derived from SPR shifted when the size of the gold nanoparticles changed. All the samples with AuPP-acridine produced gold nanoparticles of the same size, whereas all the samples produced without peptide contained gold nanoparticles of different sizes. The DLS measurements showed the same results as the UV-VIS measurements. In all samples with AuPP-acridine, gold nanoparticles after gold precipitation were the same size (approximately 10 nm, Supplementary Fig. 9a, b, c). However, the samples without AuPP-acridine had gold nanoparticles of different sizes when the sodium citrate concentration (Supplementary Fig. 9d, e, f). TEM images showed that samples with AuPP-acridine produced gold nanoparticles of the same size even when the sodium citrate concentration was changed (Supplementary Fig. 10a, b, c), whereas the samples without peptide produced gold nanoparticles of different sizes (Supplementary Fig. 10d, e, f). These results were verified using DLS and UV–VIS spectroscopy. These results imply that AuPP-acridine has the ability to take in AuCl_4_^–^.

**Fig. 3:**
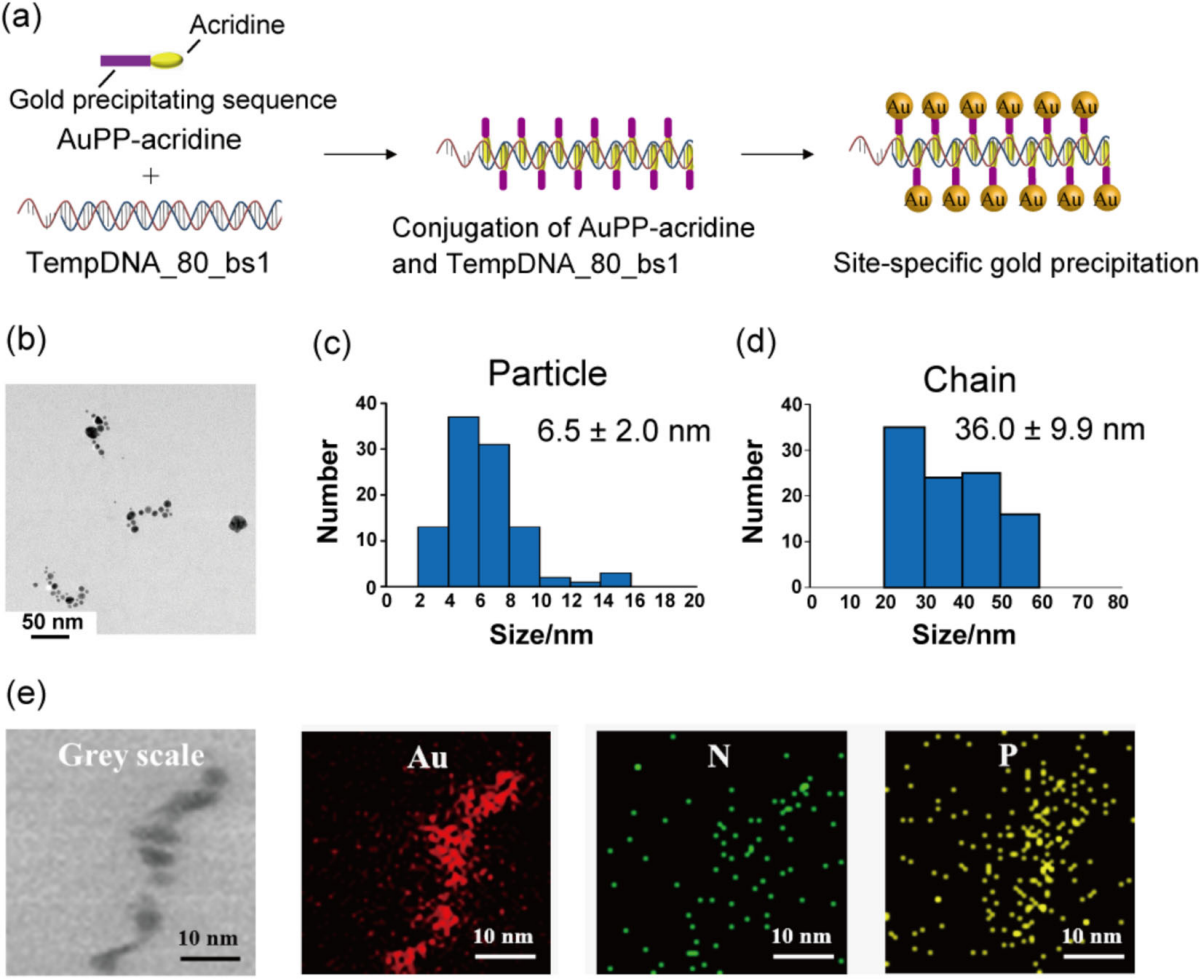
Site-specific precipitation of gold on double-strand DNA. **a** Schematic illustration of site-specific gold precipitation using AuPP-acridine and TempDNA_80_bs1. **b** TEM image of the sample after gold precipitation using AuPP-acridine and TempDNA_80_bs1. **c** Particle size distribution determined from the TEM data. **d** Chain size distribution determined from the TEM data. **e** TEM-EDX mapping images of the sample after gold precipitation using AuPP-acridine and TempDNA_80_bs1.

Then, site-specific precipitation of gold was evaluated using AuPP-acridine and TempDNA_80_bs1 (Fig. 3a). TEM observation of AuPP-acridine and TempDNA_80_bs1 without negatively stain showed a 36.0 ± 9.9 nm chain-like structure consisting of some 6.5 ± 2.0 nm diameter spherical particles (Fig. 3b, c, d). However, when using TempDNA_80_bs1 and AuPP without acridine, the TEM observation showed only spheres, similar to the results obtained with AuPP-acridine alone (Supplementary Fig. 11a, b). These results suggest that AuPP-acridine intercalates into DNA specifically and precipitates gold site-specifically on the DNA. We also investigated the elemental composition of the gold precipitation sample produced using AuPP-acridine and TempDNA_80_bs1 by TEM-EDX. Figure 3e shows a scanning TEM (STEM) image without negatively stain and several elemental EDX mapping images. Not only the gold maps, but also the nitrogen and phosphorous maps derived from peptide and DNA were correlated with the STEM image. These results suggest that both gold and peptide were present on DNA. We showed using both microscale and macroscale observations that AuPP-acridine intercalates into DNA specifically and precipitates gold site-specifically on the DNA.

### Site-specific precipitation of titania on a DNA terminus

We confirmed that titania precipitated site-specifically on DNA using TempDNA_80_bs1 and TiPP-PNA. We observed the titania precipitation conditions for subsequent UV–VIS spectroscopy (See Supplementary Methods 12 in Supplementary information) and atomic force microscopy (AFM) measurements (See Supplementary Methods 13 in Supplementary information). We then demonstrated the precipitation of titania with the peptide alone. The UV-VIS spectrum of the sample with TiPP-PNA before titania precipitation (0 h) did not show absorption by titania. However, an absorption peak derived from the anatase phase was confirmed in the sample after titania precipitation (3 h) (Supplementary Fig. 12). These results suggested that titania was precipitated within 3 h when using TiPP-PNA. Next, the morphology of the resultant titania was confirmed using AFM. TiPP-PNA provided better control of the shape of the titania particles (Supplementary Fig. 13a) than TiPP (Supplementary Fig. 13b). It was reported that a sequence consisting of hydrophobic residues such as Leu and Ile is important for the formation of a peptide assembly that will provide satisfactory shape-controlled precipitation of titania^30^. Consequently, the hydrophobicity of the PNA sequence also appears to be important for peptide assembly. These results suggest that TiPP-PNA has the ability to precipitate well-controlled titania.

**Fig. 4:**
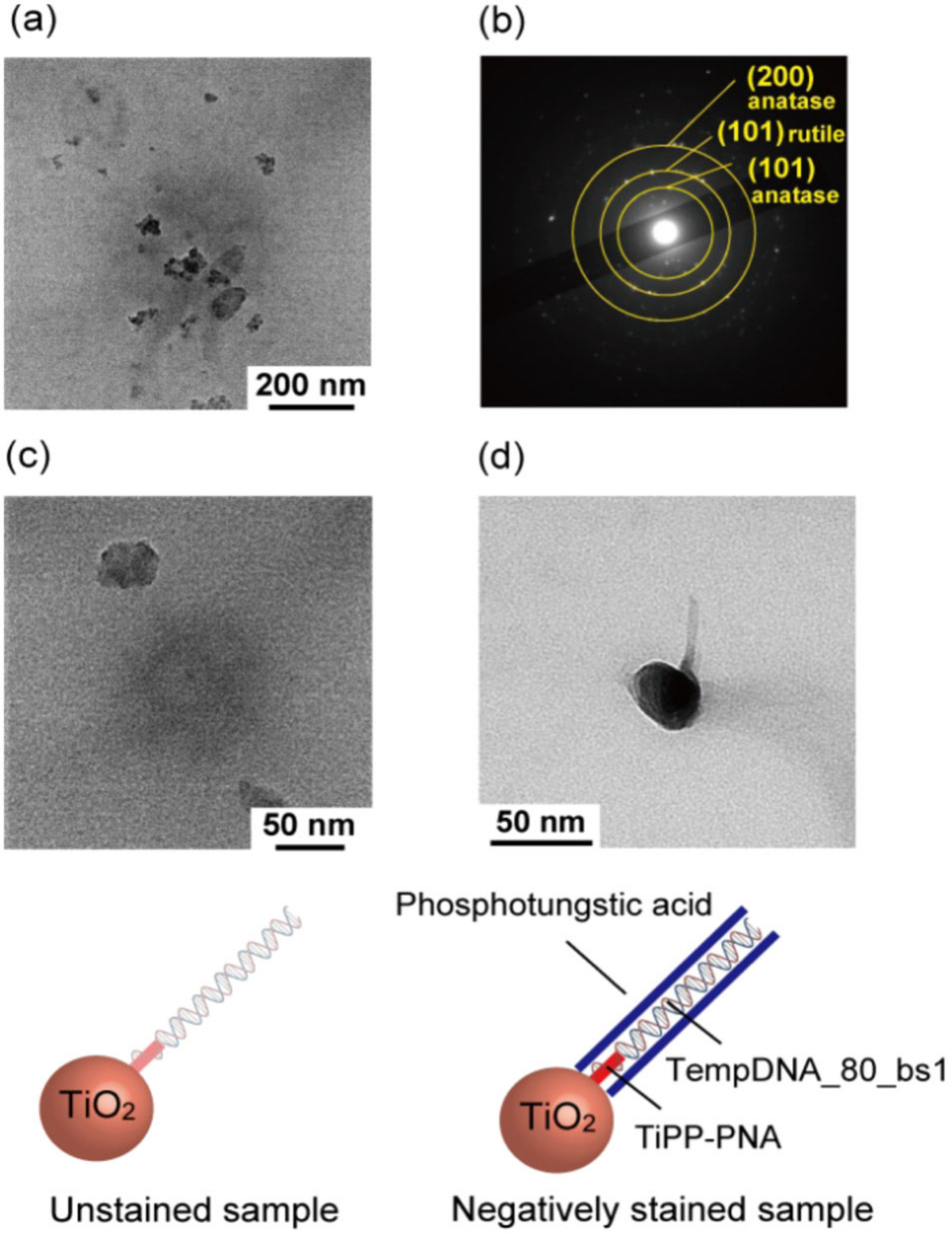
Site-specific precipitation of titania on a DNA terminus. **a** TEM image of mineralized titania after calcination at 700 °C . **b** SAED pattern of mineralized titania after calcination at 700 °C. TEM images of the sample after titania precipitation using TiPP-PNA and TempDNA_80_bs1. The TEM sample for (**c**) was not stained. The TEM sample for (**d**) was negatively stained with 2% phosphotungstic acid.

Photocatalytic activity is closely related to the crystal structure of titania. Therefore, using Raman spectroscopy and selected area electron diffraction (SAED) measurements, we identified the crystal structure of mineralized titania. We first compared the crystallinity of titania prepared by mineralization with that of commercial titania by Raman spectroscopy (See Supplementary Methods 14 in Supplementary information). The titania samples were crystallized by calcination at 700 °C (Fig. 4a). Peaks attributed to the anatase phase were detected at 144, 202, 399, 513, and 638 cm^−1^. In addition, peaks attributed to the rutile phase were detected at 240, 499, and 612 cm^−1^. The crystal phase of mineralized titania after calcination at 400 °C was mostly amorphous and partly anatase (Supplementary Fig. 14). The crystal phases of the mineralized titania after calcination at 700 °C and P25 TiO_2_ were mixed phases of anatase and rutile (Supplementary Fig. 14). These results suggest that mineralized titania has a highly crystalline structure similar to that of commercial titania. Furthermore, we identified the crystal structure of mineralized titania with peptides from the selected area electron diffraction (SAED) pattern obtained by TEM observation. The diffraction patterns of (101) and (200) derived from anatase phase and (101) derived from the rutile phase were confirmed (Fig. 4b). These results showed that mineralized titania was mixed phases of anatase and rutile and implied that the photocatalytic activity was closely related to the crystal structure of titania^46^.

We then checked the site-specific precipitation of titania using TiPP-PNA and TempDNA_80_bs1 as representative templates for comparison. AFM imaging showed that DNA and titania precipitated as chains and nanometer-sized spheres, which confirmed the successful site-specific precipitation of titania on the DNA (Supplementary Fig. 15a). In addition, using TempDNA_80_bs1 and TiPP without the PNA sequence, AFM observations revealed only spheres for titania (Supplementary Fig. 15b). The AFM observations also confirmed that TempDNA_80_bs1 alone was insufficient to precipitate titania (Supplementary Fig. 15c). When using TempDNA_80_bs1, no precipitate was observed due to the poor affinity between the DNA and mica. These results suggest that TiPP-PNA binds to DNA specifically and that this complex precipitates titania in a site-specific way.

Furthermore, the results of TEM observations were compared with those obtained by AFM. The TEM observation of an unstained sample with TiPP-PNA and TempDNA_80_bs1 revealed only nanometer-sized spheres (approximately 40 nm, Fig. 4c and Supplementary Fig. 16). However, the TEM observations of a stained sample with TiPP-PNA and TempDNA_80_bs1 showed a precipitated nanostructure consisting of short chains and nanometer-sized spheres of DNA and titania, respectively (Fig. 4d). Thus, the PNA sequence in TiPP-PNA binds to DNA specifically and this complex precipitates titania in a site-specific way.

### Detailed analysis of the photocatalytic activity of gold-titania nanocomposites

**Fig. 5:**
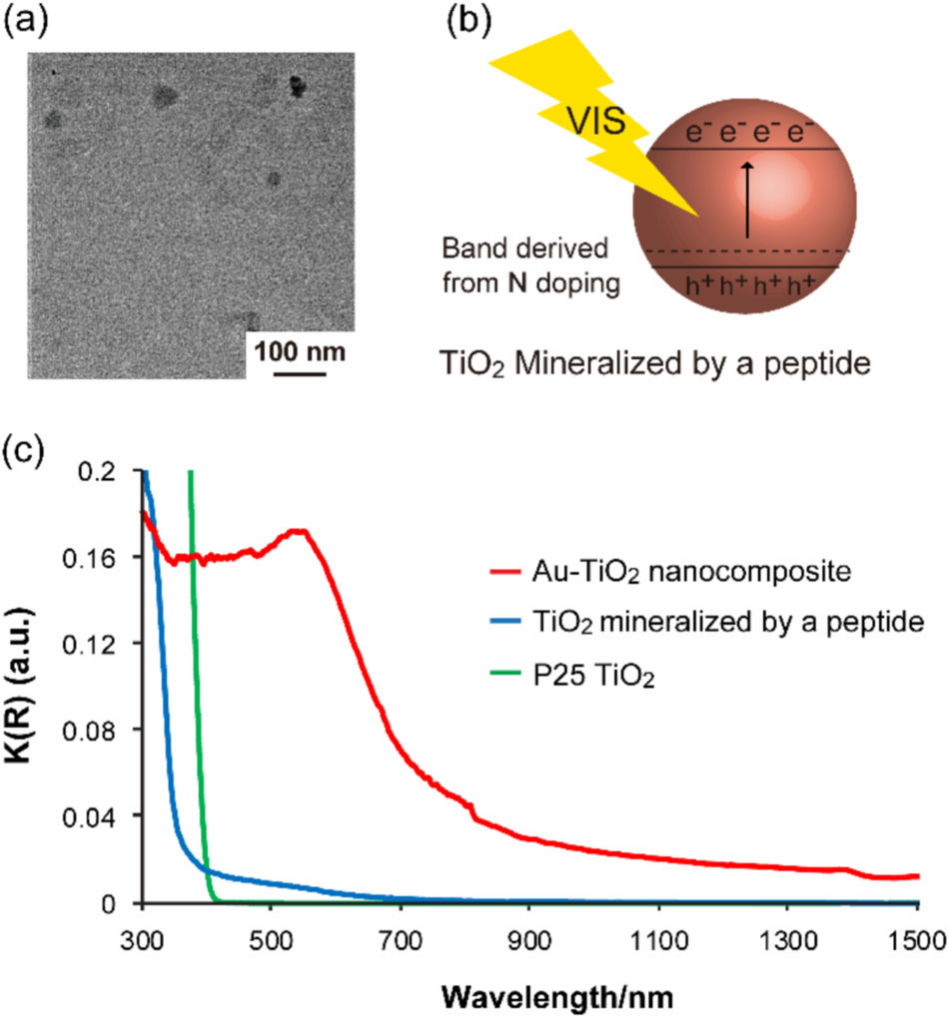
Detailed analysis of the photocatalytic activity of gold-titania nanocomposites. **a** TEM image of the sample calcined at 700 °C after titania and gold precipitation using TiPP-PNA and AuPP-acidine without TempDNA. **b** Schematic illustration of an energy diagram of titania mineralized by a peptide. **c** UV–VIS–IR DRS absorption spectra of various titania samples.

We confirmed that the gold-titania nanocomposite did not form without DNA. TEM image showed that mineralized titania and gold using two peptides without DNA dispersed, and a nanocomposite did not form (Fig. 5a). The photocatalytic activity under visible light irradiation of titania mineralized by peptides without DNA was higher than that of P25 TiO_2_ (Fig. 2e). These results imply that the peptide was completely outburned by calcination and that nitrogen was doped in the titania nanoparticles simultaneously (Fig. 5b)^46^. The photocatalytic activities of titania mineralized by a peptide without DNA and that mixed with gold mineralized by a peptide without DNA after the calcination were very low (Fig. 2e). In addition, mineralized gold with showed much lower photocatalytic activity almost the same as that of MB only (Fig. 2e). These results showed that titania and gold precipitated site-specifically on DNA and that the peptides and DNA combined as designed.

Finally, the light absorbance ability of the gold-titania nanocomposite was investigated using UV-VIS-IR diffuse reflectance spectroscopy (DRS) (See Supplementary Methods 15 in Supplementary information). Figure 5c shows the UV-VIS-IR DRS absorption spectra of the gold-titania nanocomposites produced with TempDNA_80_bs1, mineralized titania and P25 TiO_2_. Although P25 TiO_2_ showed only UV light absorption, our N-doped titania showed both UV light and visible light absorption. These results suggested that the peptide was completely outburned by calcination and that nitrogen was doped in the titania nanoparticles simultaneously (Fig. 5b)^46^. Furthermore, the gold-titania nanocomposite was confirmed to absorb over a wide range of the visible light region, and SPR derived from gold nanoparticles was observed. Thus, the gold-titania nanocomposites exhibited visible light excitation characteristics much more than that of N-doping titania.

The proposed nanometer-scale site-specific mineralization method successfully controlled the elemental composition of gold-titania nanocomposites by changing the DNA length and the number of PNA binding sites at the DNA terminus. Photocatalytic gold-titania nanocomposites responsive to visible light excitation could be constructed by our mineralization method. Furthermore, the photocatalytic activity of the gold-titania nanocomposites could be controlled by changing the gold content in the nanocomposites.

## Conclusions

Microscale techniques such as TEM, as well as macroscale techniques such as DLS and ICP-AES, were used to show that the elemental composition of a gold-titania nanocomposite can be controlled in several ten nm by changing the DNA length and the number of peptide binding sites on the DNA. Furthermore, photocatalytic activity and cell death induction effect under visible light irradiation (>450 nm) of the manufactured gold-titania nanocomposite was higher than those of commercial gold-titania (RR2Ti) and commercial titania (P25 TiO_2_). This study showed that the size, distribution, and elemental composition of nanometer-scale inorganic nanocomposites can be controlled by nanometer-scale site-specific mineralization changing the number of PNA and acridine binding sites on the DNA template. This method can be used to construct highly biocompatible functional inorganic nanocomposites without using hazardous environmental substances. The method described herein is not limited to the production of nanostructured gold and titania. This system, by substituting the titania- and gold-precipitating sequences with other inorganic compound-precipitating sequences, could provide a variety of well-controlled inorganic compound precipitations. This method could contribute to the construction of various functional materials such as chemical reaction catalysts, conductive materials, magnetic materials, and bone replacement materials. These nanocomposites produced by the site-specific precipitation method thus represent a powerful and efficient material for use in nanotechnology, electronics, ecology, medical science, and biotechnology.

## Methods

### General remarks

All chemicals and solvents were of reagent or HPLC grade and were used without further purification. Oligodeoxynucleotide PCR primers were purchased from Hokkaido System Science (Sapporo, Japan). HPLC was performed on a GL-7400 HPLC system (GL Sciences, Tokyo, Japan) using an Inertsil ODS-3 column (10 × 250 mm; GL Science) for preparative purification, with a linear acetonitrile/0.1% trifluoroacetic acid (TFA) gradient at a flow rate of 3.0 mL/min. Peptides were analyzed using MALDI-TOF MS on an Autoflex III (Bruker Daltonics, Billerica, MA, USA) mass spectrometer with 3,5-dimethoxy-4-hydroxycinnamic acid as the matrix. Amino acid analysis was conducted using an Inertsil ODS-2 column (4.6 × 200 mm; GL Science) after samples were hydrolyzed in 6 M HCl at 110 °C for 24 h in a sealed tube and then labeled with phenyl isothiocyanate.

### Preparation of gold-titania nanocomposite

Prior to preparation of the sample solutions, TempDNA and TiPP-PNA were mixed and the solvent was completely evaporated using a centrifugal evaporator (Supplementary Table 1). The sample (final conc. 10 µM) in 0.625 mM Tris-HCl buffer (pH 7.5) was then heated at 90 °C for 5 min and gently cooled to 37 °C at a rate of 1.0 °C min^−1^. The 10 µL TiPP-PNA and TempDNA solution was mixed with 10 µL of 10 mM TiBALDH solution and 90 µL of MilliQ water, and incubated for 3 h. After titania precipitation, the TiBALDH solution removed by ultrafiltration (Amicon Ultra-0.5, PLBC Ultracel-3 membrane, 3 kDa, Merck, Darmstadt, Germany). 10 µL of 200 µM AuPP-acridine was added to the ultrafiltered solution. After 30 min incubation, 10 µL of 5 mM HAuCl_4_ was added to the solution. After 30 min incubation, 10 µL of 10 mM sodium citrate was added to the solution and incubated for 12 h. After gold precipitation, HAuCl_4_ and sodium citrate were removed by ultrafiltration. A 20 µL ultrafiltered sample was placed on dried cellulose film (Cellu Sep® H1, MWCO: Norminal 2000, Funakoshi, Tokyo, Japan) and dried in vacuo. The dried cellulose film supporting the gold-titania nanocomposite was placed in an aluminum crucible and calcined. During the calcination process, the temperature was increased from 25 °C to 700 °C within 20 min. The sample was calcined for 4 h in an air atmosphere and cooled to 37 °C at a rate of 4.0 °C min^−1^. AFM, TEM, TEM-EDX, DLS, Raman spectroscopy, and UV–VIS–IR DRS were used to characterize gold-titania nanocomposite. See Supplementary information for further details of AFM, TEM, DLS, UV–VIS spectroscopy, Raman spectroscopy, ICP-AES, UV–VIS–IR DRS, cell culture, cytotoxic tests, and cell death induction experiments.

### Photocatalytic activity

The photocatalytic activities of gold-titania nanocomposite were evaluated via the decomposition of methylene blue (MB) at room temperature. The concentration of MB was set as 20 µM. The amount of titania contents in each 150 µL sample was set 0.1 mg. A 9 W tungsten halogen lamp with a cutoff filter of 450 nm was employed as the visible light irradiation source, which was located 3 cm away from the reactor to trigger the photocatalytic reaction. After recovery of the photocatalyst by centrifugation, the concentration of the dye solution was analyzed by measuring the light absorption of the clear solution at 665 nm (the λmax of the MB solution) with a UV–VIS spectrophotometer (UV-1700, Shimadzu, Kyoto, Japan).

Peptides and DNAs synthetic methods, site-specific titania precipitation method, site-specific gold precipitation method, conventional method for producing gold-titania, characterization of gold-titania nanocomposites, and corresponding data (Supplementary Fig. 1-16) are given in the Supplementary Information.

## Supplementary information

Supplementary Information

## Data Availability

The data supporting the findings of this study are available within the article and its Supplementary Information files. Other relevant source data are available from the corresponding authors upon reasonable request.

## References

[1] Chen Y (2018). Two-dimensional metal nanomaterials: synthesis, properties, and applications. Chem. Rev..

[2] Li W, Elzatahry A, Aldhayan D, Zhao D (2018). Core–shell structured titanium dioxide nanomaterials for solar energy utilization. Chem. Soc. Rev..

[3] Jana A, Scheer E, Polarz S (2017). Synthesis of graphene–transition metal oxide hybrid nanoparticles and their application in various fields. Beilstein J. Nanotechnol..

[4] Qiu T (2018). Recent advances in one-dimensional halide perovskites for optoelectronic applications. Nanoscale.

[5] Choi S, Han SI, Kim D, Hyeon T, Kim D-H (2019). High-performance stretchable conductive nanocomposites: materials, processes, and device applications. Chem. Soc. Rev..

[6] Chinnappan A, Baskar C, Kim H, Ramakrishna S (2016). Carbon nanotube hybrid nanostructures: future generation conducting materials. J. Mater. Chem. A.

[7] Tripathy N, Kim D-H (2018). Metal oxide modified ZnO nanomaterials for biosensor applications. Nano Convergence.

[8] Gai S, Li C, Yang P, Lin J (2014). Recent progress in rare earth micro/nanocrystals: soft chemical synthesis, luminescent properties, and biomedical applications. Chem. Rev..

[9] Kharlampieva E, Jung CM, Kozlovskaya V, Tsukruk VV (2010). Secondary structure of silaffin at interfaces and titania formation. J. Mater. Chem..

[10] Sumerel JL (2003). Biocatalytically templated synthesis of titanium dioxide. Chem. Mater..

[11] Crookes-Goodson WJ, Slocik JM, Naik RR (2008). Bio-directed synthesis and assembly of nanomaterials. Chem. Soc. Rev..

[12] Currie HA, Perry CC (2007). Silica in plants: biological, biochemical and chemical studies. Ann. Bot..

[13] Dickerson MB, Sandhage KH, Naik RR (2008). Protein- and peptide-directed syntheses of inorganic materials. Chem. Rev..

[14] Tomizaki K-y, Wakizaka S, Yamaguchi Y, Kobayashi A, Imai T (2014). Ultrathin gold nanoribbons synthesized within the Interior cavity of a self-assembled peptide nanoarchitecture. Langmuir.

[15] Janairo JIB, Sakaguchi T, Hara K, Fukuoka A, Sakaguchi K (2014). Effects of biomineralization peptide topology on the structure and catalytic activity of Pd nanomaterials. Chem. Commun..

[16] Tomizaki K-y, Ahn S-A, Imai T (2012). Synthesis of silica nanofibers templated by self-assembled peptide nanostructures. Trans. Mater. Res. Soc. Jpn.

[17] Ozaki M, Sakashita S, Hamada Y, Usui K (2018). Peptides for silica precipitation: amino acid sequences for directing mineralization. Protein Pept. Lett..

[18] Usui K (2018). Modification of the N-terminus of a calcium carbonate precipitating peptide affects calcium carbonate mineralization. Protein Pept. Lett..

[19] Munro CJ, Hughes ZE, Walsh TR, Knecht MR (2016). Peptide sequence effects control the single pot reduction, nucleation, and growth of Au nanoparticles. J. Phys. Chem. C..

[20] Nielsen PE, Egholm M, Berg RH, Buchardt O (1991). Sequence-selective recognition of DNA by strand displacement with a thymine-substituted polyamide. Science.

[21] Usui K, Okada A, Kobayashi K, Sugimoto N (2015). Control of guanine-rich DNA secondary structures depending on the protease activity using a designed PNA peptide. Org. Biomol. Chem..

[22] Sano S, Tomizaki K-y, Usui K, Mihara H (2006). A PNA-DNA hybridization chip approach for the detection of β-secretase activity. Bioorg. Med. Chem. Lett..

[23] Usui K (2017). DNA G-wire formation using an artificial peptide is controlled by protease activity. Molecules.

[24] Moloney GP, Kelly DP, Mack P (2001). Synthesis of acridine-based DNA bis-intercalating agents. Molecules.

[25] Jayawarna V (2006). Nanostructured hydrogels for three-dimensional cell culture through self-assembly of fluorenylmethoxycarbonyl- dipeptide. Adv. Mater..

[26] Li D (2014). Designed amyloid fibers as materials for selective carbon dioxide capture. Proc. Natl Acad. Sci. USA.

[27] Reches M, Gazit E (2003). Casting metal nanowires within discrete self-assembled peptide nanotubes. Science.

[28] Tunable assembly of amyloid-forming peptides into nanosheets as a retrovirus carrier. *Proc*. *Natl*. *Acad*. *Sci*. *USA* **112**, 2996–3001 (2015). 10.1073/pnas.1416690112 PMC4364204 25713359

[29] Makam P, Gazit E (2018). Minimalistic peptide supramolecular co-assembly: expanding the conformational space for nanotechnology. Chem. Soc. Rev..

[30] Ozaki M (2016). Site-specific control of silica mineralization on DNA using a designed peptide. Chem. Commun..

[31] Usui K (2016). Site-specific control of multiple mineralizations using a designed peptide and DNA. Nanoscale.

[32] Fujishima A, Honda K (1972). Electrochemical photolysis of water at a semiconductor electrode. Nature.

[33] Heller A (1995). Chemistry and applications of photocatalytic oxidation of thin organic films. Acc. Chem. Res..

[34] Hashimoto K, Irie H, Fujishima A (2005). TiO_2_ photocatalysis: a Historical overview and future prospects. Jpn. J. Appl. Phys..

[35] Primo A, Corma A, Garcia H (2011). Titania supported gold nanoparticles as photocatalyst. Phys. Chem. Chem. Phys..

[36] Sakamoto T, Nagao D, Noba M, Ishii H, Konno M (2014). Dispersed-nanoparticle loading synthesis for monodisperse Au-titania composite particles and their crystallization for highly active UV and visible photocatalysts. Langmuir.

[37] Tian Y, Tatsuma T (2005). Mechanisms and applications of plasmon-induced charge separation at TiO_2_ films loaded with gold nanoparticles. J. Am. Chem. Soc..

[38] Kowalska, E., Abe, R. & Ohtani, B. Visible light-induced photocatalytic reaction of gold-modified titanium(IV) oxide particles: action spectrum analysis. *Chem*. *Commun*. 241–243 (2009). 10.1039/b815679d 19099082

[39] Wen Y, Liu B, Zeng W, Wang Y (2013). Plasmonic photocatalysis properties of Au nanoparticles precipitated anatase/rutile mixed TiO_2_ nanotubes. Nanoscale.

[40] Chan, W. C. & White, P. D. Fmoc solid phase peptide synthesis; Oxford University Press: New York, NY (2000).

[41] Usui K (2009). Site-specific modification of alzheimer’s peptides by cholesterol oxidation products enhances aggregation energetics and neurotoxicity. Proc. Natl Acad. Sci. USA..

[42] Sewell SL, Wright DW (2006). Biomimetic synthesis of titanium dioxide utilizing the R5 peptide derived from Cylindrotheca fusiformis. Chem. Mater..

[43] Slocik JM, Stone MO, Naik RR (2005). Synthesis of gold nanoparticles using multifunctional peptides. Small.

[44] Random C, Wongnawa S, Boonsin P (2004). Bleaching of methylene blue by hydrated titanium dioxide. Science Asia.

[45] Kandiel TA, Feldhoff A, Robben L, Dillert R, Bahnemann DW (2010). Tailored titanium dioxide nanomaterials: anatase nanoparticles and brookite nanorods as highly active photocatalysts. Chem. Mater..

[46] Nonoyama T (2012). TiO_2_ synthesis inspired by biomineralization: control of morphology, crystal phase, and light-use efficiency in a single process. J. Am. Chem. Soc..

