# Supplementary material for: Elemental composition control of gold-titania nanocomposites by site-specific mineralization using artificial peptides and DNA
Source: Commun Chem. 2021 Jan 4;4:1. doi: 10.1038/s42004-020-00440-8 (PMC9814042; doi:10.1038/s42004-020-00440-8)
Supplement: Supplementary file 1 — Supplementary Information [file 42004_2020_440_MOESM1_ESM.pdf]

Supplementary Information

Elemental Composition Control of Gold-Titania Nanocomposites by Site-Specific Mineralization Using Artificial Peptides and DNA

Makoto Ozaki[1], Takahito Imai[2], Takaaki Tsuruoka[1], Shungo Sakashita[1], Kin-ya Tomizaki[2,3], and Kenji Usui[1]*

[1] Faculty of Frontiers of Innovative Research in Science and Technology (FIRST), Konan University, 7-1-20 Minatojima-minamimachi, Chuo-ku, 650-0047 Kobe, Japan.

[2] Department of Materials Chemistry, Ryukoku University, 1-5 Yokotani, Seta Oe-cho, 520-2194 Otsu, Japan.

[3] Department of Materials Chemistry and Innovative Materials and Processing Research Center, Ryukoku University, 1-5 Yokotani, Seta Oe-cho, 520-2194 Otsu, Japan.

E-mail: kusui@konan-u.ac.jp

CONTENTS:

Supplementary Methods;
1. Synthesis of the peptides
   1.1. Titania precipitating peptide
   1.2. Gold precipitating peptide
2. Synthesis of TempDNAs
3. Site-specific precipitation of titania on a DNA terminus
4. Site-specific precipitation of gold on double-stranded DNA
5. Inductively coupled plasma-atomic emission spectroscopy (ICP-AES)
6. Dynamic light scattering (DLS) measurements
7. Transmission electron microscopy (TEM)
   7.1. Titania precipitation sample
   7.2. Gold precipitation sample
   7.3. Gold-titania nanocomposite
8. Gold-titania generated by conventional method
9. Cell culture
10. Cell viability measurements
11. Cell death induction under visible light irradiation
12. Absorption measurements
13. Atomic force microscopy (AFM)
14. Raman spectroscopy
15. UV-VIS-IR diffuse reflectance spectroscopy (DRS)

Supplementary Table;
Supplementary Table 1

Supplementary Figures;
Supplementary Fig. 1 to Supplementary Fig. 16

Supplementary References

Supplementary Methods

1. Synthesis of the peptides

1-1. Titania precipitating peptide

Designed peptides were synthesized manually on Fmoc-NH-SAL-PEG resin (Watanabe Chemical Industries, Hiroshima, Japan) using Fmoc chemistry[1] with Fmoc-AA-OH (4 eq., Watanabe Chemical Industries, Hiroshima, Japan) and Fmoc PNA monomers (4 eq., Panagene, Daejeon, Korea) according to the *O*-(7-azabenzotriazol-1-yl)-1,1,3,3-tetramethyluronium hexafluorophosphate (HATU, Watanabe Chemical Industries, Hiroshima, Japan) method. Side-chain protection was as follows: *t*-butyl (tBu) for Ser, *t*-butyloxycarbonyl (Boc) for Lys, 2,2,4,6,7-pentamethyldihydrobenzofuran-5-sulfonyl (Pbf) for Arg, and benzhydryloxycarbonyl (Bhoc) for adenine, cytosine, guanine, and thymine PNA monomers. Peptides were cleaved from the resins and side-chain protection was removed by incubating the peptides for 1 h in TFA (Watanabe Chemical Industries, Hiroshima, Japan)/H_2O/triisopropylsilane (Fujifilm Wako Pure Chemical Industries, Tokyo, Japan) (20/1/1, v/v). Peptides were precipitated by the addition of cold diethyl ether, collected by centrifugation, purified by RP-HPLC (Supplementary Fig. 3a, b), and characterized by amino acid analysis and MALDI-TOF MS (Supplementary Fig. 3a, b): SiPP-PNA, *m/z* 3906.0 ($[M+H]^+$ calcd. 3907.1); SiPP, *m/z* 1188.9 ($[M+H]^+$ calcd. 1188.4). Purified peptides were dissolved in MilliQ water to approximately 1 mM, and their concentrations were determined by amino acid analysis. Peptides were stored at 4°C.

1-2. Gold precipitating peptide

Designed peptides were synthesized manually on TentaGel S RAM resin (HiPep, Kyoto, Japan) using Fmoc chemistry[1] with Fmoc-AA-OH (4 eq., Watanabe Chemical Industries, Hiroshima, Japan) according to the *O*-(7-azabenzotriazol-1-yl)-1,1,3,3-tetramethyluronium hexafluorophosphate (HATU, Watanabe Chemical Industries, Hiroshima, Japan) method. Side-chain protection was as follows: 4-methyltrityl (Mtt) for Lys, *t*-butyl (tBu) for Ser and Tyr. After coupling of Fmoc-Lys(Mtt)-OH, the Mtt group was removed by treatment with dichloromethane (DCM)/triisopropylsilane/TFA (94/5/1, v/v/v)[2] at 4°C. Then, acridine-9-carboxylic acid (4 eq., Fujifilm Wako Pure Chemical Industries, Tokyo, Japan) was attached to the amino group of Lys side chain according to the HATU method. After assembly of the peptides, the peptides were cleaved from the resins and side-chain protection was removed by incubating the peptides for 1 h in TFA/ethandithiol/thioanisol/m-cresol (40/3/3/1, v/v/v/v). Peptides were precipitated by the addition of cold diethyl ether, collected by centrifugation, purified by RP-HPLC (Supplementary Fig. 3c, d), and characterized by amino acid analysis and MALDI-TOF MS (Supplementary Fig. 3c, d): AuPP-acridine, *m/z* 1555.0 ($[M+H]^+$ calcd. 1555.7); AuPP, *m/z* 1221.1 ($[M+H]^+$ calcd. 1221.4). Purified peptides were dissolved in MilliQ water to approximately 1 mM, and their concentrations were determined by amino acid analysis. Peptides were stored at 4°C.

2. Synthesis of TempDNAs

TempDNA_30_bs1 consisted of TempDNA_40 (5'-TGCATCTAGTGCAATTTCTATGCGCACCCGTTCTCGGAGC-3'), which contains 1 PNA binding site, and TempDNA_30 (5'-GCTCCGAGAACGGGTGCGCATAGAAATTGC-3'). TempDNA_30_bs4 consisted of TempDNA_70 (5'-TGCATCTAGTTGCATCTAGTTGCATCTAGTTGCATCTAGTGCAATTTCTATGCGCACC CGTTCTCGGAGC-3'), which contains 4 PNA binding sites, and TempDNA_30 (5'-GCTCCGAGAACGGGTGCGCATAGAAATTGC-3'). TempDNA_80_bs1 consisted of TempDNA_90 (5'-TGCATCTAGTGCAATTTCTATGCGCACCCGTTCTCGGAGCACTGTCCGACCGCTTTGG CCGCCGCCCAGTCCTGCTCGCTTCGCTACTTG-3'), which contains 1 PNA binding site, and TempDNA_80 (5'-CAAGTAGCGAAGCGAGCAGGACTGGGCGGCGGCCAAAGCGGTCGGACAGTGCTCCG AGAACGGGTGCGCATAGAAATTGC-3').

TempDNA_150_bs1, TempDNA_300_bs1 and TempDNA_600_bs1 were amplified by PCR from pBR322 (TempDNA_150_bs1: 250-400, TempDNA_300_bs1: 250-550, TempDNA_600_bs1: 250-850). Primers used for amplification of TempDNA_150_bs1 were (5'-ACGTAGATCAGCAATTTCTATGCGCACCCGTTCTC-3') and (5'-CACGATGCGTCCGGCGTAGAG-3'). Primers used for amplification of TempDNA_300_bs1 were (5'-ACGTAGATCAGCAATTTCTATGCGCACCCGTTCTC-3') and (5'-GCCCAACAGTCCCCCGGCCAC-3'). Primers used for amplification of TempDNA_600_bs1 were (5'-ACGTAGATCAGCAATTTCTATGCGCACCCGTTCTC-3') and (5'-GAATACCGCAAGCGACAGGCC-3'). After PCR, the samples were purified using a MinElute PCR Purification Kit (QIAGEN, Tokyo, Japan).

3. Site-specific precipitation of titania on a DNA terminus

Prior to precipitation of the sample solutions, TempDNA and TiPP-PNA were mixed and the solvent was completely evaporated using a centrifugal evaporator. Next, each sample (final conc. 10 μM) in 0.625 mM Tris-HCl buffer (pH 7.5) was heated at 90°C for 5 min and then gently cooled to 37°C at a rate of 1.0°C min^{-1}. Prior to titania precipitation, 100 mM titanium bis(ammonium lactato)dihydroxyde (TiBALDH) was prepared by mixing TiBALDH and MilliQ water and incubating for 5 min, and then diluting the solution 10 times with MilliQ water. Finally, 1 μL of TiPP-PNA and TempDNA solution was mixed with 1 μL of 10 mM TiBALDH solution and 9 μL of MilliQ water, and incubated for 3 h. Samples incubated for 3 h were mixed with 9 μL of MilliQ water.

4. Site-specific precipitation of gold on double-stranded DNA

2 μL of 200 μM AuPP-acridine was mixed with 2 μL of 10 μM TempDNA_80_bs1 and 12 μL of MilliQ water, and incubated for 30 min. After 30 min incubation, 2 μL of 5 mM $HAuCl_4$ was added to the solution. After 30 min incubation, 2 μL of 10 mM sodium citrate was added to the solution and incubated for 12 h.

5. Inductively coupled plasma-atomic emission spectroscopy (ICP-AES)

Titania samples were pyrolyzed with 2.5 mL of (1+1) aqua regia, 2.5 mL of $HClO_4$ and 5 mL of MilliQ water for 1 h at 250°C. After pyrolysis, samples were dissolved with 2.5 mL of (1+1) aqua regia and 22.5 mL of MilliQ water. Calibration curves for each element were obtained using gold standard solution (for Atomic Absorption Spectrochemical Analysis, Fujifilm Wako Pure Chemical Industries, Tokyo, Japan) and titanium standard solution (for Atomic Absorption Spectrochemical Analysis, Fujifilm Wako Pure Chemical Industries, Tokyo, Japan) in the range of 0 ppm to 10 ppm. The ratios of gold to titanium were detected at wavelengths of 242.795 nm (gold) and 389.785 nm (titanium) using ICP-AES (SPECTROBLUE® FMX36 provided by Clean Chemical Co. Ltd., Hitachi, Tokyo, Japan).

6. Dynamic light scattering (DLS) measurements

The sample solution (40 μL) was transferred into a UV-transparent disposable cuvette (S3, Sarstedt, Germany), and DLS data were acquired on a Zetasizer ZEN3600 instrument (Sysmex, Kobe, Japan) equipped with a 633-nm laser.

7. Transmission electron microscopy (TEM)

7-1. Titania precipitation sample

Samples incubated for 3 h were mixed with 9 μL of MilliQ water, and the entire volume of each sample was placed on a TEM grid (Cu 200 mesh covered with a collodion membrane, Nisshin EM, Tokyo, Japan) for 1 min and dried with filter paper. MilliQ water (20 μL) was then placed on the grid and immediately absorbed with filter paper. This process was repeated three times to remove salts from the sample. All samples were dried *in vacuo* prior to TEM measurements, which were conducted at an accelerating voltage of 200 kV (JEM-2100, JEOL, Tokyo, Japan). Stain samples were negatively stained with 2 % phosphotungstic acid.

7-2. Gold precipitation sample

Prior to gold precipitation, the gold precipitation sample solution (20 μL) was incubated for 12 h and then placed on a TEM grid for 1 min and dried with filter paper. MilliQ water (20 μL) was then

placed on the grid and immediately absorbed with filter paper. This process was repeated three times to remove salts from the sample. All samples were dried *in vacuo* prior to TEM measurements, which were conducted at an accelerating voltage of 200 kV (JEM-2100, JEOL, Tokyo, Japan).

7-3. Gold-titania nanocomposite

The gold-titania sample (20 µL) was placed on a TEM grid for 1 min and dried with filter paper. MilliQ water (20 µL) was then placed on the grid and immediately absorbed with filter paper. This process was repeated three times. All samples were dried *in vacuo* prior to TEM measurements, which were conducted at an accelerating voltage of 115 kV (JEM-1400, JEOL, Tokyo, Japan).

8. Gold-titania generated by conventional method

15 mL of 10 mM TiBALDH and 15 mL of 10 mM urea were added to 120 mL of MilliQ water, and incubated for 24 h at 160°C. Titania generated by centrifuging at 20,000 g for 30 min was recovered, and dried *in vacuo*[3]. 40 mL of 5 mM $HAuCl_4$ was mixed with 340 mL MilliQ water, and 20 mL of 20 mM $NaBH_4$ was added to 380 µL of $HAuCl_4$ solution. Incubated for 30 min at r.t. Gold nanoparticles generated by centrifuging at 20,000 g for 30 min were recovered, and dried *in vacuo*. The dried titania and gold were placed in an aluminum crucible and calcined. During the calcination process, the temperature was increased from 25°C to 700°C within 20 min. The sample was calcined for 4 h in an air atmosphere and cooled to 37°C at a rate of 4.0°C min^{-1}.

9. Cell culture

Human cervical carcinoma (HeLa) cells were cultured in Dulbecco's modified Eagle's medium (D-MEM) supplemented with 10% fetal bovine serum and 1% penicillin streptomycin. HeLa cells were seeded with 2.5×10^4 cells in 24-well plate (Nest Biotechnology Co. Ltd., USA) and cultured at 37°C in a 5% CO_2 atmosphere for 24 h.

10. Cell viability measurements

HeLa cells were seeded with 2.5×10^4 cells in a 24-well plate and cultured at 37°C in a 5% CO_2 atmosphere for 24 h. 500 µL of 0.1 mg/mL titania sample in D-MEM were added to 24-well plates and incubated at 37°C in a 5% CO_2 atmosphere for 24 h. After incubation, the media were removed and the cells were washed three times with 1×PBS. The cells were then treated with 200 µL of 0.25% trypsin at 37°C in a 5% CO_2 atmosphere for 3 min. After adding 300 µL of the media, the cells were transferred into a microtube. The media were removed by centrifugation at 1,500 rpm for 5 min (room temperature). Cells were suspended in 50 µL of 1×HEPES, and transferred into a 96-well plate. 1×HEPES (40 µL) and Cell Counting Kit-8 (CCK-8, 10 µL, Dojindo Molecular

Technologies, Tokyo, Japan) were added to the cell suspensions and kept at 37°C in a 5% CO_2 atmosphere for 1 h. The UV signal was monitored at 450 nm using a UV spectrophotometer (MTP-310 Microplate Reader, Colona Electric, Ibaraki, Japan).

11. Cell death induction under visible light irradiation

HeLa cells were seeded with 5.0×10^4 cells in a 24-well plate and cultured at 37°C in a 5% CO_2 atmosphere for 24 h. 500 µL of 0.1 mg/mL gold-titania samples in PBS were added to 24-well plates, and HeLa cells were irradiated with visible light (>450 nm) for 20 min. After incubation, the media were removed and the cells were washed three times with 1×PBS. The cells were then treated with 200 µL of 0.25% trypsin at 37°C in a 5% CO_2 atmosphere for 3 min. After adding 300 µL of the media, the cells were transferred into a microtube. The media were removed by centrifugation at 1,500 rpm for 5 min (room temperature). Cells were suspended in 50 µL of 1×HEPES, and transferred into a 96-well plate. 1×HEPES (40 µL) and CCK-8 (10 µL) were added to the cell suspensions and kept at 37°C in a 5% CO_2 atmosphere for 1 h. The UV signal was monitored at 450 nm using a UV spectrophotometer (MTP-310 Microplate Reader, Colona Electric, Ibaraki, Japan).

12. Absorption measurements

The sample solution (130 µL) was transferred into a quartz cuvette with a 1 cm pathlength. All absorption spectra data were acquired on a UV-1800 spectrometer (Shimadzu, Kyoto, Japan).

13. Atomic force microscopy (AFM)

The entire volume of each sample was placed on freshly cleaved mica (1 x 1 cm). After 5 min, the solvent was absorbed with filter paper. MilliQ water (20 µL) was then placed on the mica surface and immediately absorbed with filter paper. This process was repeated three times to remove salts from the sample. All samples were dried *in vacuo* prior to AFM measurements. Tapping-mode images were obtained on a MultiMode scanning probe microscope with a Nanoscope IIIa controller (Veeco, Woodbury, NY, USA).

14. Raman spectroscopy

Raman spectra were obtained with a confocal Raman spectrometer (RMP-510, JASCO, Tokyo, Japan). Mineralized titania by peptide was calcined with same protocol as that of the gold-titania nanocomposite and then the titania samples were placed on the stage of the spectrometer. The collection time was 10 s and the ranges of Raman shift were between 100 cm^{-1} and 1000 cm^{-1}.

15. UV-VIS-IR diffuse reflectance spectroscopy (DRS)

The UV-VIS-IR absorption spectra were measured under the diffuse reflection mode using an integrating sphere (ISR-240A, Shimadzu, Kyoto, Japan) attached to a UV-VIS-IR spectrophotometer (SolidSpec-3700 provided by Hyogo Prefectural Institute of Technology, Shimadzu, Kyoto, Japan), and $BaSO_4$ (Fujifilm Wako Pure Chemical Industries, Tokyo, Japan) was used as a reference. Titania samples were pressed to form a pellet and placed on a $BaSO_4$ plate.

Supplementary Table

Supplementary Table 1 Gold and titania mineralization conditions in this study.

DNA *1	TempDNA_30_bs4	TempDNA_30_bs1	TempDNA_80_bs1	TempDNA_150_bs1	TempDNA_300_bs1	TempDNA_600_bs1
TiPP-PNA binding sites	4	1	1	1	1	1
AuPP-acridine intercalating sites	10	10	20	50	100	200
TiPP-PNA concentration *2	4 μM	1 μM	1 μM	1 μM	1 μM	1 μM
AuPP-acridine concentration *2	10 μM	10 μM	20 μM	50 μM	100 μM	200 μM

*1 Gold-titania nanocomposites were manufactured at DNA concentration of 1 μM under all conditions.

*2 The amounts of these peptides in each batch of mineralization reaction were adjusted to the numbers of the peptide binding sites of used DNA.

Supplementary Figures

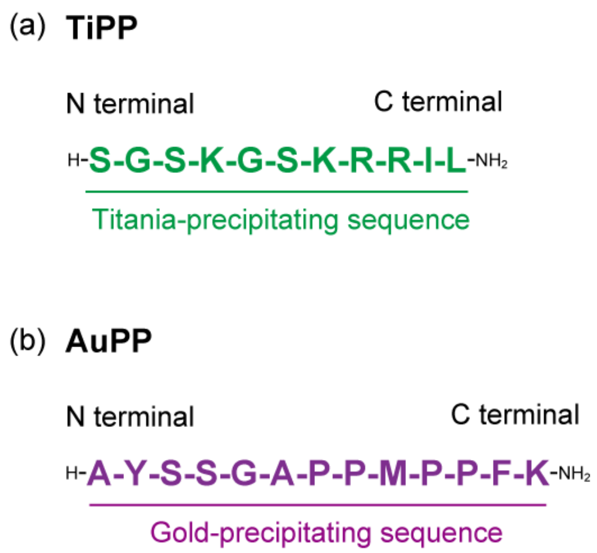

Supplementary Fig. 1 Sequences of (a) titania precipitating peptide (TiPP) and (b) gold precipitating peptide (AuPP).

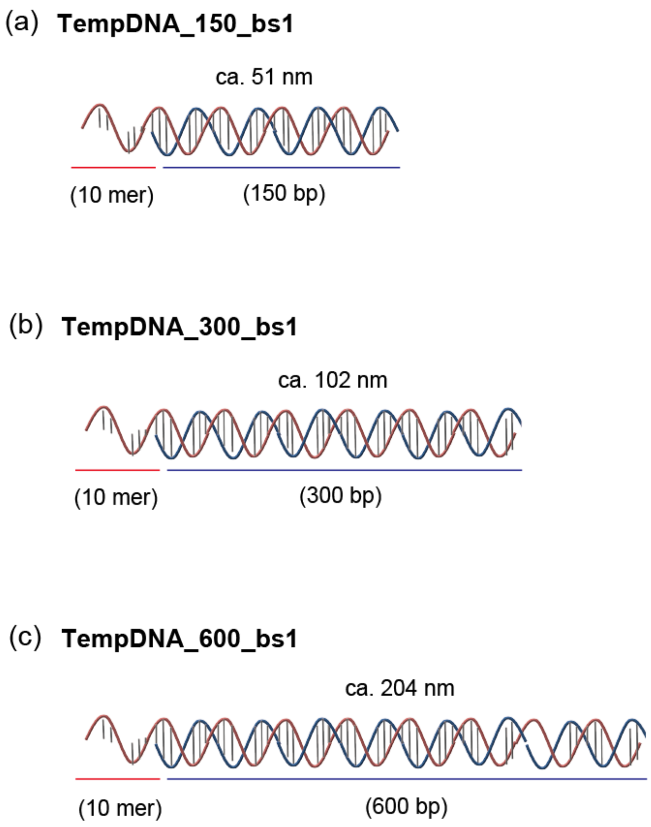

Supplementary Fig. 2 Illustration of (a) TempDNA_150_bs1, (b) TempDNA_300_bs1, and (c) TempDNA_600_bs1 used in this study.

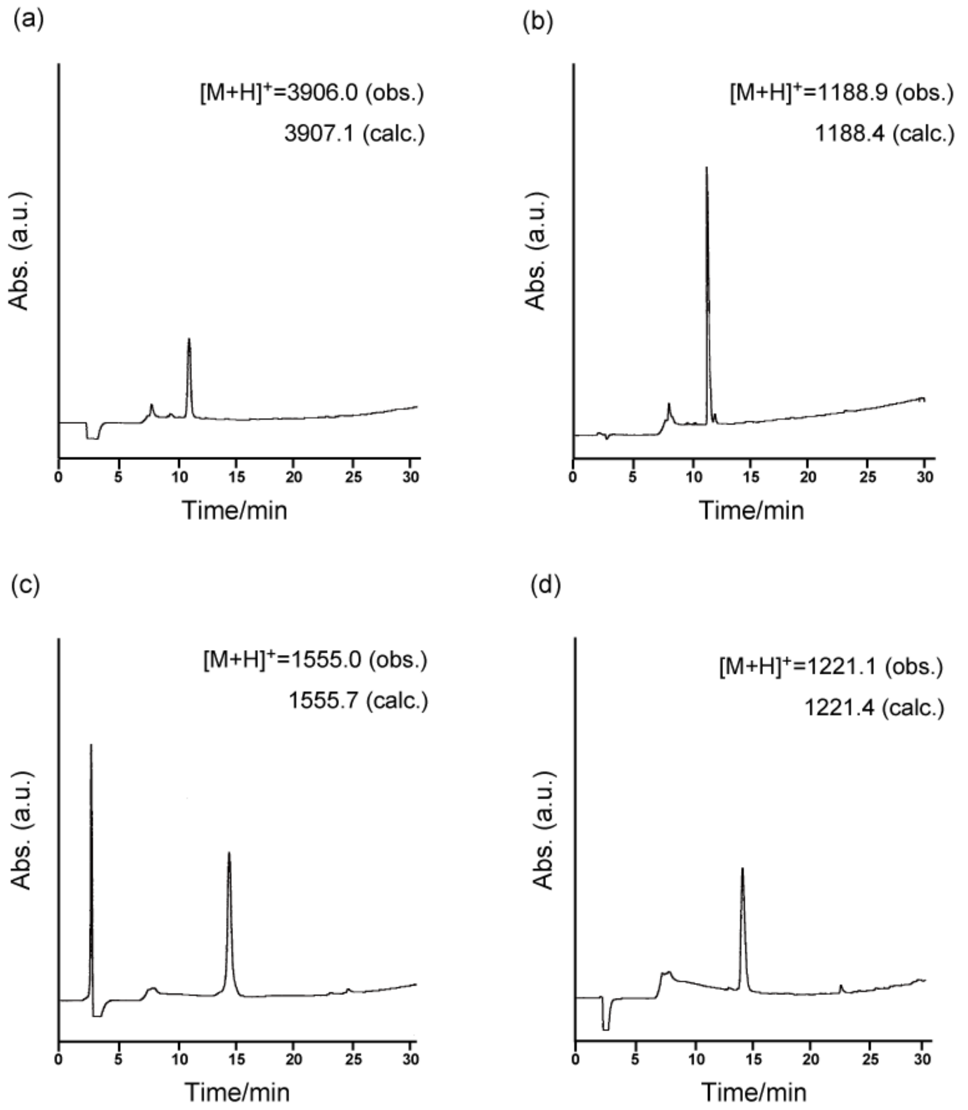

Supplementary Fig. 3 HPLC for purified (a) TiPP-PNA, (b) TiPP, (c) AuPP-acridine, and (d) AuPP separated on an ODS columun (150×4.6 mm, 5 µm) with MilliQ water (containing 0.1% TFA) using a gradient from 0% to 100% acetonitrile (containing 0.08% TFA) over 30 min, 1.0 mL/min; detection time at 220 nm.

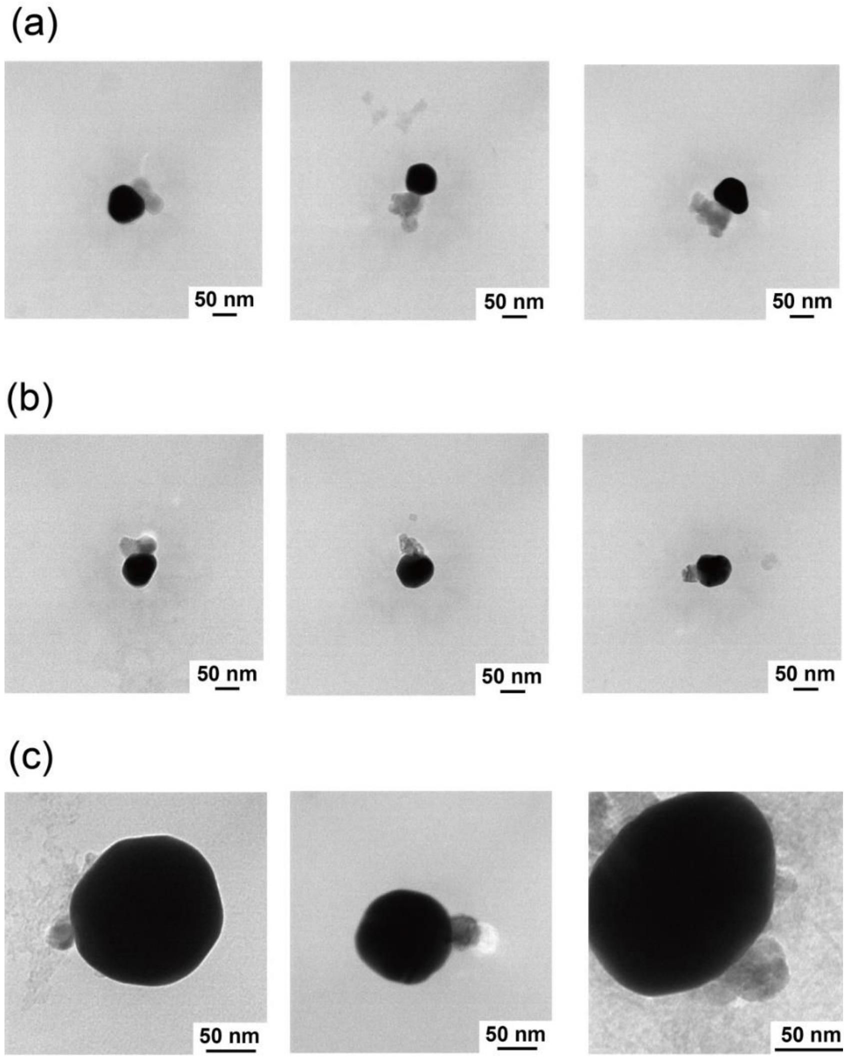

Supplementary Fig. 4 TEM images of gold-titania nanocomposites with (a) TempDNA_30_bs4, (b) TempDNA_30_bs1, and (c) TempDNA_80_bs1.

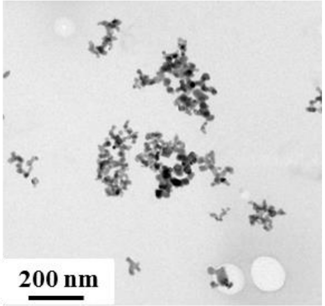

Supplementary Fig. 5 TEM image of commercial titania (P25 TiO_2).

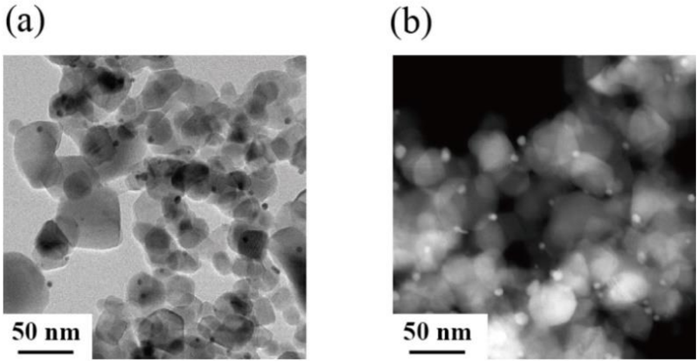

Supplementary Fig. 6 (a) TEM image and (b) HAADF-STEM image of commercial gold-titania (RR2Ti). These TEM data were supplied by Haruta Gold Co. Ltd.

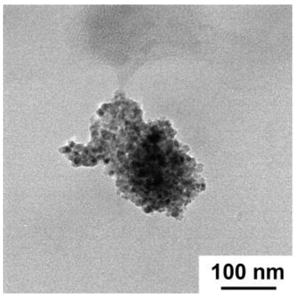

Supplementary Fig. 7 TEM image of gold-titania generated by conventional method

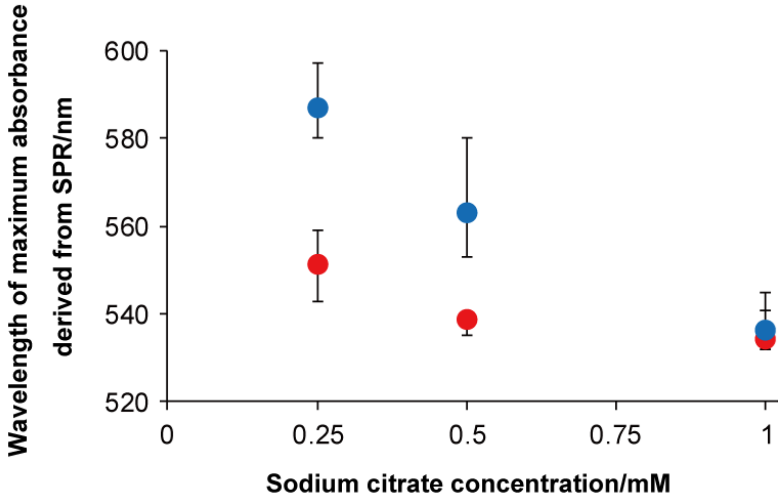

Supplementary Fig. 8 UV-VIS data for the samples after gold precipitation with(red) / without(blue) AuPP-acridine with sodium citrate at 1 mM, 500 µM, and 250 µM.

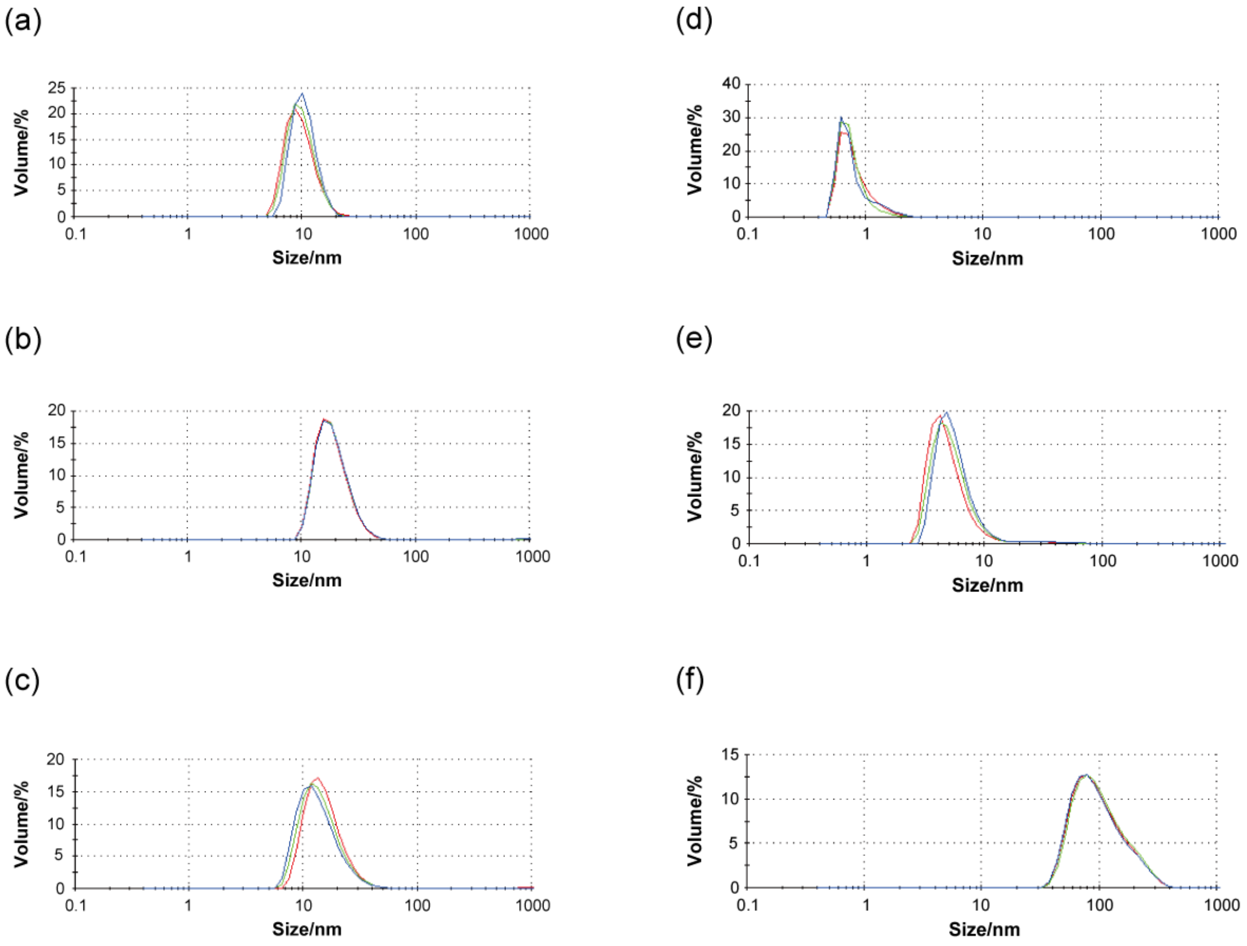

Supplementary Fig. 9 DLS data for the samples after gold precipitation using AuPP-acridine with sodium citrate at (a) 1 mM, (b) 500 µM, and (c) 250 µM. DLS data for the samples after gold precipitation without AuPP-acridine but with sodium citrate at (d) 1 mM, (e) 500 µM, and (f) 250 µM.

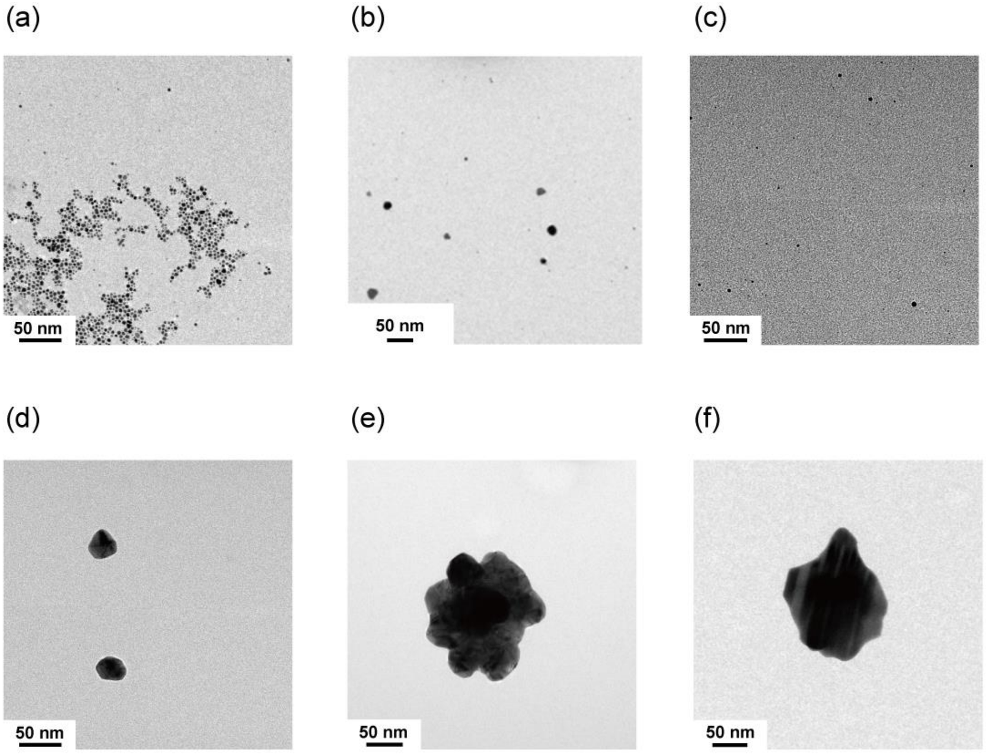

Supplementary Fig. 10 TEM images of the samples after gold precipitation using AuPP-acridine with sodium citrate at (a) 1 mM, (b) 500 µM, and (c) 250 µM. TEM images of gold precipitation without AuPP-acridine but with sodium citrate at (d) 1 mM, (e) 500 µM, and (f) 250 µM.

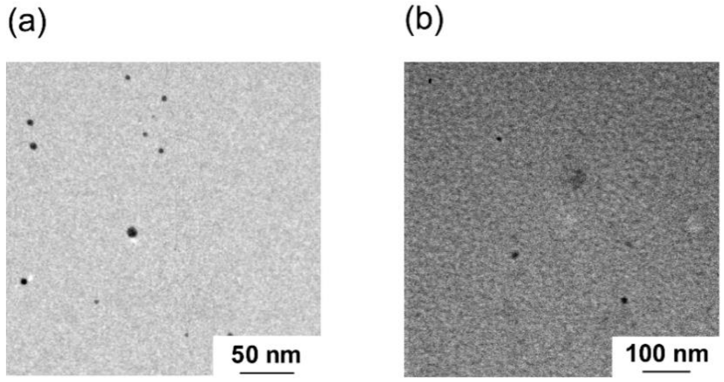

Supplementary Fig. 11 TEM images of the samples after gold precipitation using (a) TempDNA_80_bs1 and AuPP (without acridine moiety), and (b) AuPP-acridine with sodium citrate at 1 mM (without TempDNA).

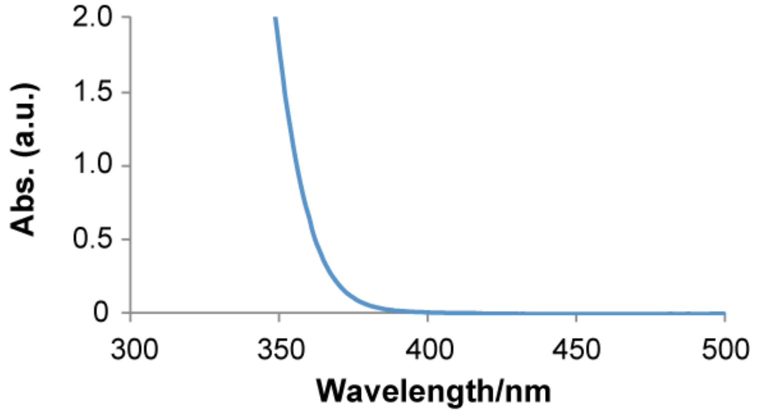

Supplementary Fig. 12 UV-VIS spectrum of the sample after titania precipitation using TiPP-PNA.

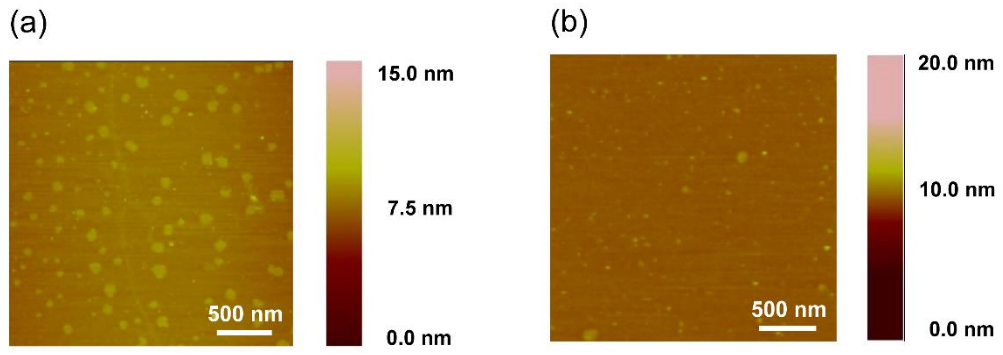

Supplementary Fig. 13 AFM images of the sample after titania precipitation using (a) TiPP-PNA and (b) TiPP.

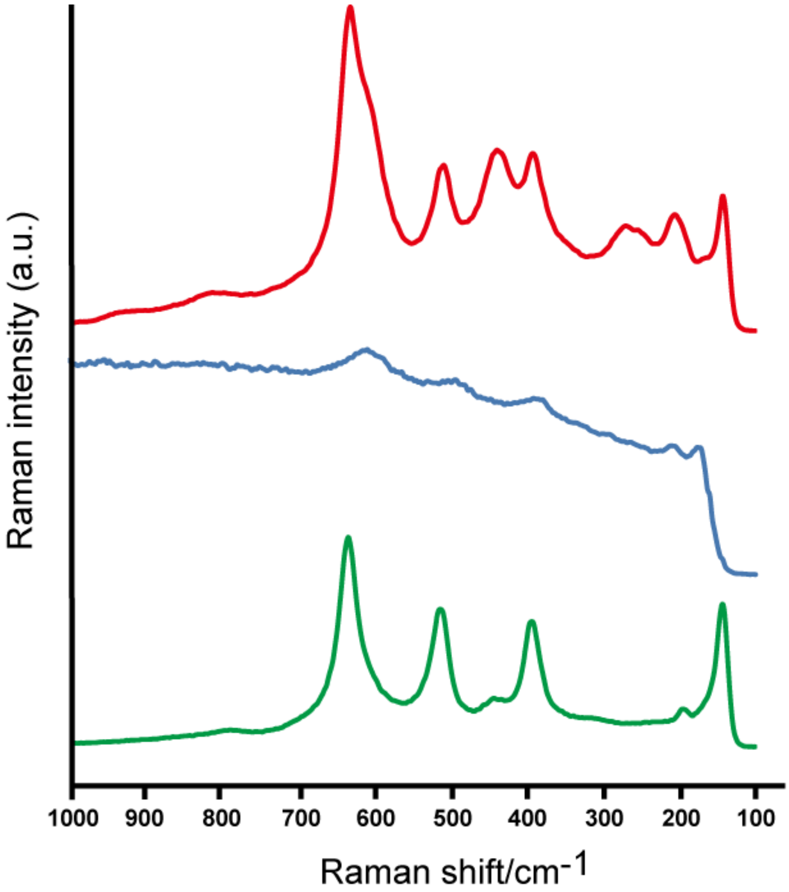

Supplementary Fig. 14 Raman spectra of various titania samples. The red line represents titania mineralized by a peptide after calcinated at 700°C. The blue line represents titania mineralized by a peptide after calcinated at 400°C. The green line represents P25 TiO_2.

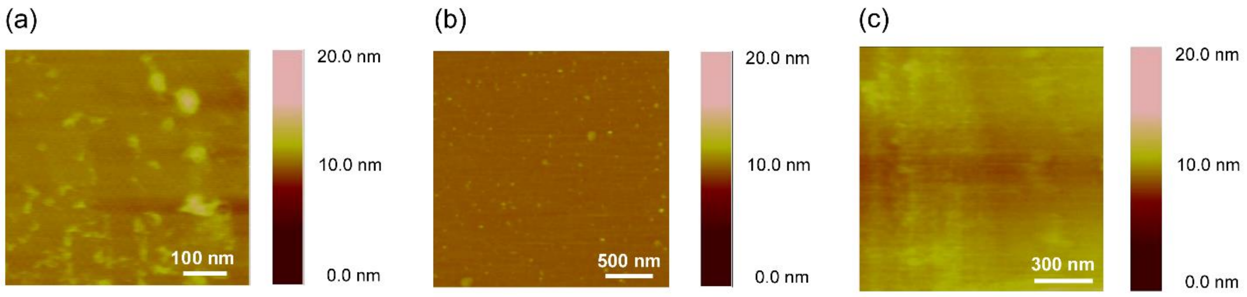

Supplementary Fig. 15 AFM images of the sample after titania precipitation using (a) TempDNA_80_bs1 and TiPP-PNA, (b) TempDNA_80_bs1 and TiPP, and (c) TempDNA_80_bs1 alone.

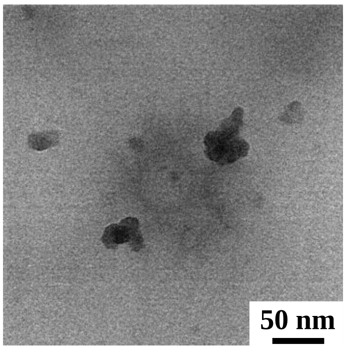

Supplementary Fig. 16 TEM image of the sample after titania precipitation using TempDNA_80_bs1 and TiPP-PNA.

Supplementary References

1. Chan, W. C. & White, P. D. Fmoc Solid Phase Peptide Synthesis; Oxford University Press: New York, 2000.
2. Usui, K., Hulleman, J. D., Paulsson, J. F., Siegel, S. J., Powers, E. T. & Kelly, J. W. Site-specific modification of alzheimer's peptides by cholesterol oxidation products enhances aggregation energetics and neurotoxicity. *Proc. Natl. Acad. Sci. USA.* **106**, 18563-18568 (2009).
3. Kandiel, T. A., Feldhoff, A., Robben, L., Dillert, R. & Bahnemann, D. W. Tailored titanium dioxide nanomaterials: anatase nanoparticles and brookite nanorods as highly active photocatalysts. *Chem. Mater.* **22**, 2050-2060 (2010).
